# The Gut Mycobiome for Precision Medicine

**DOI:** 10.3390/jof11040279

**Published:** 2025-04-02

**Authors:** Islam El Jaddaoui, Sofia Sehli, Najib Al Idrissi, Youssef Bakri, Lahcen Belyamani, Hassan Ghazal

**Affiliations:** 1 Laboratory of Human Pathologies Biology, Department of Biology, Faculty of Sciences, University Mohammed V, Rabat 10000, Morocco; is.eljaddaoui@gmail.com (I.E.J.); y.bakri@um5r.ac.ma (Y.B.); 2 Genomic Center of Human Pathologies, Faculty of Medicine and Pharmacy, University Mohammed V, Rabat 10000, Morocco; 3 Laboratory of Precision Medicine & One Health (MedPreOne), School of Medicine, Mohammed VI University of Sciences & Health, Casablanca 82403, Morocco; ssehli@um6ss.ma (S.S.); nalidrissi@um6ss.ma (N.A.I.); 4 School of Medicine, Mohammed VI University of Sciences & Health, Casablanca 82403, Morocco; belyamani@gmail.com; 5 Laboratory of Sports Sciences and Performance Optimization, Royal Institute of Executive Management, Salé 10102, Morocco; 6 National Center for Scientific and Technical Research, Rabat 10102, Morocco

**Keywords:** gut mycobiome, precision medicine, NGS, human diseases

## Abstract

The human gastrointestinal tract harbors a vast array of microorganisms, which play essential roles in maintaining metabolic balance and immune function. While bacteria dominate the gut microbiome, fungi represent a much smaller, often overlooked fraction. Despite their relatively low abundance, fungi may significantly influence both health and disease. Advances in next-generation sequencing, metagenomics, metatranscriptomics, metaproteomics, metabolomics, and computational biology have provided novel opportunities to study the gut mycobiome, shedding light on its composition, functional genes, and metabolite interactions. Emerging evidence links fungal dysbiosis to various diseases, including inflammatory bowel disease, colorectal cancer, metabolic disorders, and neurological conditions. The gut mycobiome also presents a promising avenue for precision medicine, particularly in biomarker discovery, disease diagnostics, and targeted therapeutics. Nonetheless, significant challenges remain in effectively integrating gut mycobiome knowledge into clinical practice. This review examines gut fungal microbiota, highlighting analytical methods, associations with human diseases, and its potential role in precision medicine. It also discusses pathways for clinical translation, particularly in diagnosis and treatment, while addressing key barriers to implementation.

## 1. Introduction

**Figure 1:**
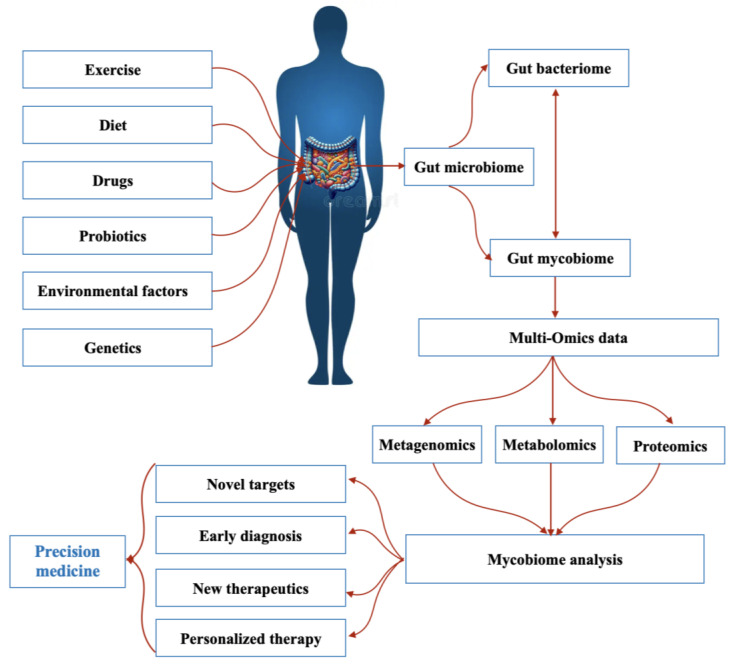
The gut mycobiome and precision medicine.

In addition to bacteria and viruses, the human gut hosts a diverse fungal community that plays a crucial role in maintaining intestinal balance and influencing disease development [1]. Over the past decade, advancements in next-generation sequencing (NGS) have significantly expanded our understanding of gut mycobiome diversity, revealing numerous fungal species that contribute to health and disease [2]. By improving microbial classification, NGS has propelled research on microbial interactions, microbiome–disease correlations, and host–microbiota relationships [3]. Simultaneously, the field of precision medicine has emerged, aiming to deliver personalized healthcare based on an individual’s genetic profile, lifestyle, medical history, and environmental factors [4]. The integration of microbiome research into precision medicine holds great potential for improving diagnostic accuracy, minimizing treatment risks, and reducing adverse drug reactions. These advancements could ultimately lead to a more cost-effective, prevention-oriented healthcare model [5] (Figure 1). As recognition of the role of the gut microbiome in precision medicine has increased, interest in the mycobiome as a potential target for personalized therapies has increased.

Despite its promise, mycobiome-based diagnostics and treatments face significant challenges, primarily due to the lack of standardized methodologies [6]. Mycobiome compositions vary widely among individuals and can undergo rapid changes, making it a complex and dynamic field of study. While these variations offer opportunities for highly personalized treatments, achieving precision microbial medicine will require further research to fully understand the functional roles of gut fungi [5]. Nonetheless, ongoing scientific efforts continue to advance our knowledge of the mycobiome, fostering optimism for the development of mycobiome-based diagnostic tools and therapeutic strategies. In this review, we explore the fungal microbiota of the human gut, discuss the analytical techniques used for its study, and examine its potential links to human diseases. Additionally, we highlight the role of the mycobiome in precision medicine and discuss challenges that must be addressed to translate these findings into clinical applications, particularly in disease diagnosis and treatment.

## 2. The Gut Mycobiome

Microbial communities, including fungi, bacteria, and archaea, are referred to as microbiota, and the genes they encode are known as the microbiome [7]. Although the makeup and dynamics of the microbiome’s bacterial elements have been extensively studied in health and illness, other microbiome members are far less well understood. Currently, little is known about the mycobiome and virome, the fungal and viral components of the microbiome, and their involvement in the development of health and illness in humans is poorly characterized [8,9]. Metagenomic analyses of the gut microbiota have shown that more than 99% of detected genes belong to bacteria, whereas fewer than 0.1% originate from eukaryotic or viral sources [10]. Although fungi constitute only a minor fraction of the gut microbiota (0.1%) [11], their relatively larger size and unique mechanisms of interaction with human tissues and the immune system suggest a distinct role in maintaining host health or contributing to disease [12,13]. Although some substantial research has focused on the gut mycobiome, a universally accepted definition of a “healthy gut mycobiome” remains elusive. A healthy gut mycobiome refers to the diverse yet balanced fungal community within the human gastrointestinal tract that interacts with bacteria and the immune system to support homeostasis and overall health. It is typically dominated by commensal yeasts such as *Saccharomyces cerevisiae*, *Malassezia restricta*, and *Candida albicans*, which coexist with bacteria to aid digestion, modulate immune responses, and prevent pathogen overgrowth [14]. The study of Nash et al. (2017) [1] analyzed the human gut mycobiome using stool samples from the Human Microbiome Project (HMP) healthy cohort, with a focus on fungal diversity and composition. Using Internal Transcribed Spacer 2 (ITS2) and 18S rRNA gene sequencing, researchers found that fungal diversity was significantly lower than bacterial diversity, with yeast dominating the samples. The most prevalent genera were *Saccharomyces*, *Malassezia*, and *Candida*, particularly *S. cerevisiae*, *M. restricta*, and *C. albicans*, which were found in the majority of samples. While fungal communities exhibited high inter- and intra-individual variability, some species persisted across multiple samples, suggesting the presence of a core gut mycobiome [1].

Factors such as temporal fluctuations in fungal communities, their low abundance and diversity, and significant variation among individuals contribute to this lack of consensus [1,15]. Nonetheless, a healthy microbiome exhibits a set of shared characteristics that can be distinguished from those of unhealthy individuals. Therefore, understanding the differential properties of the microbiome may aid in the detection and identification of disease-associated mycobiomes [7].

### 2.1. Gut Mycobiome Composition

A total of at least 267 distinct fungal taxa have been identified in the gut, which is significantly lower than the bacterial taxa present therein [16]. The human gut microbiome is highly diverse, with a core microbiome (~200 common species) and a variable component (~800+ species depending on geography, diet, and health). However, the concept of a “core gut mycobiome” remains debated, and it is unclear whether such a classification applies to fungal communities [17]. To date, numerous studies have documented a wide variety of fungal genera in the gut. Diverse fungal communities in all sections of the human gut have been revealed, consisting mainly of the phyla Zygomycota, *Basidiomycota*, Chytridiomycota, and Ascomycota [18,19]. The species *Aspergillus versicolor*, *Candida* spp., *Cladosporium herbarum*, *Cryptococcus* spp., *Debaryomyces hansenii*, *Galactomyces Geotrichum*, *Malassezia* spp., *Penicillium commune*, *Saccharomyces cerevisiae*, and *Trichosporon* spp. have been frequently reported from the gut [16,20].

### 2.2. Gut Mycobiome Determinants of Variation

Compared with the gut bacterial microbiome, the gut mycobiome exhibits greater variability between individuals and greater instability over time [21,22]. Various factors, including ethnicity, urbanization, geographic location, dietary habits, and lifestyle, are key determinants shaping the composition of the gut mycobiota [22,23]. In addition to dietary and environmental influences, host factors such as genetics, sex, age, and medication also contribute to gut mycobiome variation [24]. Like the overall microbiome, the mycobiome in infants is highly variable during the first year of life and is significantly influenced by birth mode, breastfeeding practices, diet, and age. Throughout life, diet continuously impacts the intestinal microbiome, as the human gut is constantly exposed to food-borne microbes [13,25]. While shifts in the intestinal bacteriome are driven by nutrient availability, the mycobiome is shaped by food colonizers and the ingestion of environmental fungi [26,27].

### 2.3. Gut Mycobiome Functions

The gut microbiota is widely recognized for its diverse beneficial functions, including protection against pathogens, facilitation of nutrient absorption, energy metabolism, synthesis of vitamins and cofactors, detoxification of xenobiotics, and regulation of immune system development and function. It also plays a crucial role in the gut–brain axis [28]. However, disruptions in the composition and functionality of the gut microbiota can lead to dysbiosis [10]. Imbalances within the gut mycobiota have been linked to various diseases, including autoimmune, metabolic, and neurological disorders, as well as cancers. The overgrowth and colonization of opportunistic fungal pathogens in the gut can trigger abnormal immune responses, ultimately affecting disease progression [22]. Understanding the role of the mycobiome in health and disease is crucial for several reasons. First, the prevalence of fungal infections has increased significantly in recent decades, with fungi increasingly being linked to well-established chronic illnesses. Second, growing evidence indicates that fungi are key players in modulating the host immune response and influencing disease progression through complex fungal–fungal, fungal–bacterial, and fungal–host interactions [29]. Collectively, these interactions between gut fungi, bacteria, and the host immune system form the foundation of immune homeostasis, ultimately shaping both health and disease outcomes [22].

The human mycobiome serves as a key diagnostic biomarker, with increasing research focusing on its potential therapeutic applications [30]. Each organ in the human body harbors a distinct mycobiota, contributing uniquely to disease processes. Understanding variations in the mycobiome among individuals, as well as the distribution and frequency of fungal populations across different organs, provides valuable insights into disease development. Since the mycobiome influences various clinical outcomes, individual differences in drug responses may also be attributed to the mycobiome composition. Therefore, incorporating mycobiome-based personalized medicine—alongside genetic profiling—could significantly increase drug efficacy and play a crucial role in modern healthcare strategies [7].

## 3. Methodologies to Study the Gut Mycobiome

### 3.1. Culture-Dependent Methods

Traditional approaches for investigating the mycobiome have relied on culture-based techniques. These methods commonly involve classic microbiological procedures, such as microscopy, biochemical tests, and/or cultivation on selective media [31]. In studies focusing on the gut mycobiome, researchers often conduct serial dilutions of fecal samples and plate them onto media enriched with antibiotics [32]. These plates are then used to quantify fungi and/or isolate colonies for further analysis. A variety of commercially available media are used for examining the gastrointestinal mycobiome, in addition to those that can be prepared via basic techniques [31]. Commercially available media, such as potato dextrose and Sabouraud dextrose, are the most commonly used. More specialized fungal media, such as Dixon’s and Czapek–Dox media, have been employed in efforts to isolate more challenging fungal species from the human gut [33]. However, traditional culturing techniques are prone to several inherent challenges and biases. The difficulty in culturing the majority of fungal microorganisms within the microbiome mirrors similar challenges faced when attempting to culture bacterial and archaeal species [28]. Many species are undetected due to the lack of known optimal culture conditions, and the culture process itself is time intensive and not suitable for high-throughput applications [2]. In culture-based research, the presence of dominant populations, such as *Candida* species, can obscure the detection and diversity of less abundant organisms. Moreover, existing fungal culture techniques may be insufficient to effectively cultivate species that rely on microbe–microbe interactions, as they fail to replicate the ecological niches and symbiotic relationships found in natural environments [16]. The culturable fraction of the microbiome is also skewed due to differences in replication rates between microorganisms, with fast-growing species often outcompeting slower-growing ones. Nevertheless, culture-dependent methods are still valuable and of significant interest. Additionally, several studies have demonstrated that some strains are identifiable using culture-dependent methods but not through culture-independent approaches. This suggests that current DNA extraction or PCR amplification techniques may not be optimal, potentially leading to the underrepresentation of certain strains, possibly owing to unique cell wall structures or specific DNA sequences. As a result, many research groups combine both culture-dependent and culture-independent methods to increase the identification of fungal diversity [2].

### 3.2. Culture-Independent Methods

#### 3.2.1. Metagenomics

The process of characterizing the microbiome, including the mycobiome, begins with sample collection, which presents several challenges in fungal analysis. First, fungi are often found in much lower abundance than bacteria in human-associated environments. The abundance of both fungi and bacteria can be compared by analyzing metagenomic sequencing data, where the relative abundance is determined by counting the sequence reads corresponding to each organism type. Second, the presence of human cells can introduce contamination, potentially hindering the specific amplification and sequencing of fungal DNA. Last, extracting high-quality genetic material from fungi can be more difficult than extracting it from bacteria or animal cells. These factors must be carefully considered when designing sample collection protocols [28].

For the human intestinal mycobiome, feces are most frequently used as a convenient and noninvasive sample type from which to obtain an overview of the types of organisms present in the intestinal tract [34]. Various DNA extraction techniques have been employed to isolate total microbial and fungal DNA from feces. However, the rigid structure of fungal cells poses a challenge, often affecting both DNA yield and quality owing to the difficulty of cell lysis. While some commercial kits effectively extract total DNA, they may not efficiently recover fungal DNA, potentially leading to an underestimation of fungal presence in the gut. To address this, modifications such as bead beating and enzymatic, fungal-specific lysis steps have been incorporated into commercial DNA extraction kits [31].

Advancements in NGS have significantly enhanced metagenomic research, making it more accessible and cost-effective for the scientific community. Metagenomics refers to the genetic analysis of uncultured microbial communities from environmental samples and relies on sequence-based methods and bioinformatics tools. These studies focus on determining the taxonomic diversity of the microbiota—identifying both the number and types of microbes in a community—or exploring their functional roles through functional metagenomics [35]. Two primary approaches are used: marker gene analysis and shotgun sequencing. Marker gene analysis involves designing primers that target highly conserved regions of specific genes, such as the internal transcribed spacer (ITS) for fungi, to establish phylogenetic relationships within microbial communities.

Most fecal mycobiome studies rely on marker or amplicon sequencing for strain identification. Fungal rRNA genes contain highly conserved regions that serve as primer binding sites, enabling the amplification and sequencing of adjacent variable regions through PCR [16]. A commonly targeted locus for fungal amplicon sequencing is the rRNA gene region [36], which consists of the ribosomal small subunit (18S) and large subunit (26S) and is separated by the ITS regions ITS1 and ITS2 [37]. While the fungal 18S rRNA gene, similar to the bacterial 16S rRNA gene, has historically been used for amplicon-based studies, it is more suitable for distinguishing higher taxonomic ranks. In contrast, the ITS regions exhibit significant sequence variability, making them highly effective for differentiating fungal genera [13]. The choice of the ITS target region remains crucial, as primer selection can introduce biases. For example, the ITS1 and ITS1-F primers tend to favor Basidiomycetes, whereas the ITS2 and ITS4 primers are more suitable for amplifying Ascomycetes [38].

ITS amplicon sequencing involves selectively amplifying and sequencing a small genomic region of fungal DNA, followed by alignment with reference sequences of known identity [39]. Various sequencing technologies, including the Roche 454, Illumina HiSeq-MiSeq, and Ion Torrent methods, have been employed to process diverse amplicons generated via PCR across multiple samples. These platforms generate massive amounts of sequencing data, ranging from millions to trillions of reads, and require efficient storage and computational analysis. To aid in taxonomic classification and comparative analyses of sample compositions across study groups, an increasing number of reference databases and bioinformatics tools have been developed [28].

As an alternative approach, microbiome shotgun sequencing enables a comprehensive and unbiased analysis of the entire mycobiome without relying on amplicon-based methods [39]. This technique involves sequencing all extracted DNA from a sample without the need for targeted PCR amplification of the internal transcribed spacer (ITS) region or other specific markers. Shotgun sequencing provides in-depth genetic and taxonomic insights into the gut microbiota but is more costly and time-consuming than marker gene analysis is. Additionally, while shotgun sequencing can infer potential functions of microbial communities at the gene level using reference genome and metabolic pathways databases, it may not accurately reflect real-time microbial activity in the gut. This limitation arises because the method captures DNA from all microbes present at the time of sampling, including metabolically active, dormant, and nonviable organisms [40].

Characterizing the mycobiome is still challenging due to the limited availability of comprehensive, well-curated, and high-resolution taxonomic annotations in fungal databases. Several databases contain fungal reference sequences, including UNITE, ITSoneDB, Findley, RefSeq targeted loci, ITS2 database IV, the targeted host-associated fungi (THF) database, the International Society for Human and Animal Mycology (ISHAM) ITS database, and SILVA [41]. Additionally, the Ribosomal Database Project (RDP) is widely used for fungal taxonomic classification [41]. To facilitate mycobiome analysis, existing bioinformatics tools originally developed for bacterial and archaeal studies have been adapted for fungal community profiling. Commonly used software programs include MEGAN, QIIME, MetaPhlAn, and mothur [26].

Our knowledge of the fecal microbiota is largely influenced by the strengths and limitations of current methodologies. Various methodological factors can impact fecal mycobiome analysis outcomes, and no universally accepted approach has been established. This uncertainty applies to all stages of research, from sample collection and sequencing techniques to data analysis and interpretation. Therefore, careful consideration is necessary throughout the process to account for methodological differences and minimize potential biases that could distort the results [39].

#### 3.2.2. Other Omic Techniques

To gain a comprehensive understanding of the role of the human mycobiome in health and disease, metagenomics should be complemented by other meta-omic approaches. These techniques help determine whether mycobiome alterations are a cause or a consequence of disease [42]. Metatranscriptomics, for example, focuses on sequencing microbial mRNAs to identify actively expressed genes within a microbial community, revealing how gene expression fluctuates in response to microenvironmental conditions [35]. Since mRNAs constitute less than 5% of a cell’s total RNA, detecting and quantifying gut microbial mRNAs provides valuable insights into the genes and pathways that are functionally active in the gut ecosystem and influence health and disease [40]. A key limitation of metatranscriptomics is the short half-life of messenger RNA, which decays rapidly—often within minutes—meaning that the results may not accurately reflect the microbial activities occurring in situ [43].

Metaproteomics focuses on identifying, quantifying, and determining the potential functions of microbial proteins [44]. This approach uses high-resolution mass spectrometry to detect and quantify proteins that are expressed within a microbial community. The data generated are processed through analytical pipelines, which match the resulting peptides to metagenomic databases to identify the microbes likely responsible for protein expression.

Finally, metabolomics focuses on identifying and quantifying the small-molecule metabolites produced by microbes under specific physiological and environmental conditions. This helps to reveal the dynamic metabolic functions of microbial communities and how they impact their human host [35]. Two approaches can be used: targeted and untargeted metabolomics. In targeted metabolomics, the focus is on quantifying metabolites involved in specific pathways related to particular diseases. In contrast, untargeted metabolomics aims to measure as many metabolites as possible from samples without any preconceived bias [40]. This approach typically involves isolating metabolites from body samples, such as urine, feces, or blood, and analyzing them using technologies such as nuclear magnetic resonance (NMR) or microscopy–mass spectrometry. The results are spectral sequences that correspond to the specific absorption patterns of the metabolites present in the sample [43].

## 4. Aspects of Fungal Pathogenesis

### 4.1. Fungal Virulence Factors

Virulence results from a complex interplay between the infecting microorganism and the host, with pathogenesis depending on interactions between multiple factors on both sides. This is especially evident in fungal pathogenesis, where the specific mechanisms remain less understood compared to bacterial pathogens. Unlike bacteria, only a few fungi function as obligate pathogens. The majority exist as saprophytes in environmental niches or as commensals within the human microbiota, typically without causing harm. However, certain fungi have the capacity to infect even healthy individuals, leading to severe systemic diseases. Due to the intricate nature of host–fungus interactions, only a limited number of factors are absolutely essential for fungal virulence. These include the ability to grow at elevated temperatures, adherence to host tissues, penetration and dissemination within the host, and efficient acquisition of nutrients, all of which contribute to fungal survival and pathogenic potential [45].

*C. albicans* is a natural component of the human microbiome but has the ability to cause infections ranging from mild superficial conditions to severe, life-threatening systemic diseases. Its virulence is largely attributed to its exceptional metabolic adaptability and its ability to transition between yeast and hyphal forms. This yeast-to-hypha dimorphic switch is accompanied by a genetic program that enables key pathogenic traits, including adhesion to host surfaces, thigmotropism (directional growth in response to touch), active and induced invasion of host cells, acquisition of essential nutrients, direct damage to host tissues, biofilm formation, and multiple immune evasion strategies. In contrast, *C. glabrata* lacks true hyphal formation yet remains a formidable pathogen. Its success in causing infections is primarily due to its high intrinsic resistance to environmental stresses and its ability to evade the host immune response, making it a significant opportunistic fungal pathogen [46].

*Cryptococcus neoformans* and *C. gattii* are facultative intracellular yeasts with several virulence factors that contribute to their pathogenicity. One of their most notable features is the production of a thick polysaccharide capsule, which plays a crucial role in immune evasion. This capsule suppresses cytokine production by immune cells, sequesters complement components to interfere with immune signaling, and reduces the antigen-presenting capacity of monocytes, effectively shielding the fungus from detection. By masking itself from phagocytes, *Cryptococcus* species can avoid being engulfed and destroyed. In addition to capsule formation, other significant virulence traits include melanin production, which provides protection against oxidative stress and enhances survival in hostile environments, the ability to thrive at human body temperature (37 °C), and the secretion of extracellular enzymes that facilitate tissue invasion and nutrient acquisition [46].

### 4.2. Interactions with the Host Immune System

The immune system is continuously exposed to various fungal species and has developed multiple defense mechanisms to counter pathogenic fungi. A primary line of defense includes mucosal membranes, such as those lining the gastrointestinal and respiratory tracts. These epithelial barriers are integral components of the innate immune system. While certain fungi exist as commensals in these regions without causing harm, fungal infections, or mycoses, typically arise when the integrity of these protective barriers is compromised [31].

The innate immune system plays a vital role in recognizing a wide range of microbes by detecting distinct microbial signatures, such as lipoproteins and microbial DNA. Host cells equipped with pattern recognition receptors (PRRs), including those within the innate immune system and various epithelial cells, can identify fungal cell wall components, which contain specific structural polysaccharides like chitin, mannan, and β-glucan. These fungal elements act as pathogen-associated molecular patterns (PAMPs) and are recognized by the host immune system. This recognition triggers the innate immune response, activating multiple intracellular signaling pathways that initiate proinflammatory and antimicrobial reactions. Consequently, gene expression is induced, leading to the production of cytokines, chemokines, cell adhesion molecules, and immune receptors, all of which contribute to coordinating the adaptive immune response against fungal pathogens. Ultimately, this microbial recognition process facilitates the development of adaptive immunity [31].

β-Glucan is a key structural component of the fungal cell wall and interacts with Dectin-1, a type II transmembrane receptor that possesses a single extracellular C-type lectin domain. As a result, Dectin-1 is classified as a C-type lectin receptor (CLR) and functions by binding to β-glucans. This interaction plays a crucial role in the immune response by facilitating the phagocytosis of fungal cells by macrophages and contributing to the production of reactive oxygen species, which aid in fungal clearance [16].

### 4.3. Production of Metabolites

Similar to bacteria, fungi can produce metabolites that impact host homeostasis and exert biological effects as part of fungi–host interactions. Certain fungal species, including *Saccharomyces* boulardii, *S. cerevisiae*, and *Candida albicans*, secrete regulatory molecules such as farnesol, fusel alcohols, tyrosol, and fatty acids. These molecules play a role in modulating fungal growth by influencing processes like adhesion, yeast-to-hypha transition, and biofilm formation, ultimately aiding in colonization, invasion, and dissemination within the host. Additionally, β-1,3-glucan, a polysaccharide found in the inner cell wall of *C. albicans*, is another fungal-derived molecule involved in host interactions [15].

Candidalysin is a peptide toxin secreted by *C. albicans* that functions as a proinflammatory molecule, contributing to both local and systemic infections. It plays a crucial role in immune activation by directly inducing the production of proinflammatory cytokines such as IL-1α, IL-1β, IL-8, and IL-36. Additionally, candidalysin stimulates the activation of the NOD-, leucine-rich repeat (LRR)-, and pyrin domain-containing protein 3 (NLRP3) inflammasome, which is a key regulator in inflammatory processes associated with metabolic disorders, including obesity, type 2 diabetes (T2DM), and nonalcoholic fatty liver disease (NAFLD) [47].

Studies have shown that *Candida* parapsilosis, *Cryptococcus neoformans*, *Candida albicans*, and *Meyerozyma guilliermondii* are capable of producing prostaglandins (PGs) from exogenous arachidonic acid. Notably, PGs derived from M. guilliermondii have been found to reach the liver and exacerbate alcoholic hepatic steatosis in mice [48]. Additionally, *C. albicans*-derived prostaglandin E2 (PGE2) is believed to influence host immune responses during infection [49]. Furthermore, fungal species such as *Saccharomyces* and *Candida* have been reported to produce ethanol in the gastrointestinal tract, potentially leading to auto-brewery syndrome, also known as gut fermentation syndrome [47].

## 5. The Gut Mycobiome in Disease

Investigating fungal communities in various disease conditions can provide valuable insights into causality and disease progression. Table 1 presents an analysis of 82 studies examining gut mycobiome dysbiosis linked to 56 human diseases. In total, dysbiosis within the gut mycobiome involves 102 distinct fungal genera. Among these, *Candida* is the most frequently associated with disease, followed by *Saccharomyces*, *Aspergillus*, and *Malassezia*. Other implicated genera, though less common, include *Penicillium*, *Exophiala*, and *Rhodotorula*.

Disruptions in the gut mycobiome have been strongly linked to intestinal and metabolic disorders, as well as cancer progression. Additionally, research suggests its involvement in diseases affecting the liver; kidneys; skin; eyes; and gynecological, cardiovascular, and respiratory systems. Furthermore, significant shifts in gut fungal composition have been observed in infectious diseases, as well as in immunological, neurological, and genetic conditions, among others (Table 1). In terms of geographical locations, research on the gut mycobiome has been conducted in a diverse range of countries. Notably, a significant portion of the studies (36 out of 82) were carried out in various locations across China, with additional research conducted in other countries in North America, South America, Europe, Asia, and Africa.

These findings highlight the widespread influence of gut fungi on the host’s systemic health, and observational studies examining gut mycobiome alterations in patients consistently indicate a connection between fungal dysbiosis and disease development. However, the relationship between fungal imbalances and disease progression is complex, as dysbiosis may act as both a contributing factor and a consequence, depending on the condition. Therefore, more comprehensive research is needed to clarify the specific roles of fungi in various diseases and explore their potential clinical applications.

The studies we present herein provide valuable insights into fungal dysbiosis in a wide range of human diseases. However, inconsistencies can be observed across studies, particularly due to methodological differences in sampling, DNA extraction, sequencing technique and depth, and fungal identification methods, with the absence of standardized protocols for mycobiome analysis. Many studies rely heavily on ITS sequencing for fungal identification, which can miss or misclassify species, and there is a notable lack of comprehensive data regarding the functional roles of specific fungi in disease pathogenesis. Similarly, few studies have relied on integrative multi-omics approaches, which integrate various omic profiles to reveal complex host–microbe interactions. Moreover, the studies also present limitations related to relatively small and geographically restricted cohorts, which may limit their generalizability.

The discrepancies in findings can also be a result of biases in data interpretation that may arise from the focus on certain fungal taxa (e.g., *Candida*) while neglecting others, potentially leading to an incomplete picture of the role of the gut mycobiome in disease. Furthermore, the cross-sectional nature of many studies limits causal inferences, making it unclear whether observed changes are causal or secondary to disease. Cross-sectional research also neglects potential confounders such as diet, genetics, and medication use, which are often insufficiently controlled. Geographic and demographic variability, short follow-up periods in some cases, and the challenge of distinguishing transient vs. stable fungal colonizers further hinder reproducibility. Addressing these constraints through larger, more rigorous, longitudinal studies with standardized methodologies will be essential to fully elucidate the role of the mycobiome in diseases and eventually translate mycobiome research into clinical practice.

### 5.1. Dysbiosis of the Gut Mycobiome in Gastrointestinal Tract Diseases

The evidence resulting from gut microbiota analyses and immune responses to GI fungi suggests a potential link between fungi and inflammatory bowel disease (IBD). Chehoud et al. (2015) [50] showed that *Candida* was more common in stool samples from children with IBD than in those from healthy children via ITS1 region gene sequencing [50]. Sokol et al. (2017) [51] also reported distinct fungal microbiota dysbiosis in the stool of patients with IBD characterized by a decreased proportion of *S. cerevisiae* and increased *C. albicans* using ITS2 sequencing [51]. *S. cerevisiae* was also found to be significantly more prevalent in IBD patients in the study of Yu et al. (2023) [52]. However, the abundance of *Saccharomyces*, in addition to Sarocladium, decreased in IBD patients in the study of Imai et al. (2019) [53], which was conducted in a Japanese population [53]. Overall, the gut fungal microbiota is altered in IBD, but how fungi are involved in the occurrence and development of IBD remains debatable. Like the findings of the majority of studies examining IBD, irritable bowel syndrome has also been found to be characterized by a high abundance of *Candida* [54]. Nonetheless, diarrhea-predominant irritable bowel syndrome has a distinct fungal profile characterized by an increase in four genera, namely, *Mycosphaerella*, *Aspergillus*, *Sporidiobolus*, and *Pandora* [55].

In addition to IBD, the gut mycobiome has also been implicated in Crohn’s disease (CD) and ulcerative colitis (UC). Qiu et al. (2020) [56] assessed the prevalence of fungal taxa in CD patients and compared the results to those of healthy subjects. Accordingly, they presented an increased number of *Candida* and decreased numbers of *Aspergillus*, *Sordariomycetes*, and *Penicillium* in CD patients [56]. Similarly, Li et al. (2014) [57] reported an increased abundance of *C. albicans*, *Aspergillus clavatus*, and C. *neoformans* species and suggested that the gut mycobiome shifts with inflammation and disease severity in CD patients [57]. *Candida* species were also markedly elevated in CD patients in studies carried out by Hoarau et al. (2016) [58], Krawczyk et al. (2021) [59], and Krawczyk et al. (2023) [60]. However, there was a contradiction regarding *Malassezia*. The latter was enriched in CD patients in the study of Krawczyk et al. (2023) [60] and depleted in the work of Krawczyk et al. (2021) [59]. Furthermore, Zeng et al. (2022) [61] recently reported in a study of a southwestern Chinese population that *Exophiala dermatitidis*, *Clonostachys*, *Humicola*, and *Lophiostoma* were more abundant in CD patients [61].

UC, another gastrointestinal tract disease, has also been explored to detect any fungal signatures in patients. Azizollah et al. (2024) [62] studied an Iranian cohort and reported that both *Candida* and *Saccharomyces* proportions decreased in patients [62]. Other unique genera, including *Scytalidium*, *Morchella*, *Paecilomyces* [63], *Piptoporus*, and *Hyphodontia* [64], are increased in UC patients.

### 5.2. Dysbiosis of the Gut Mycobiome in Metabolic Diseases

Accumulating evidence has shown that patients with metabolic diseases such as diabetes, obesity, and NAFLD have distinct gut fungal dysbiosis, and differences in the gut fungal mycobiome between patients and healthy controls have been identified (Table 1). Studies have shown that gut mycobiome homeostasis is disrupted in patients with type 1 diabetes (T1DM). Honkanen et al. (2020) [65] reported that *Candida* and *Saccharomyces* are enriched [65]; conversely, Salamon et al. (2021) [66] reported that *Saccharomyces* was depleted in T1DM patients [66]. This variability across studies might be attributed to different assay methods and sample sizes. Gut mycobiome dysbiosis has also been documented in T2DM patients. Some gut fungi, such as *Agaricus*, *Chlorophyllum*, *Coprinopsis*, *Leucoagaricus*, *Termitomyces*, *Trametes*, *Trichoderma*, *Volvariella* [67], and unclassified *Basidiomycota* [68], were clearly decreased, whereas *Candida*, *Aspergillus* [69], *Malessezia firfur*, and unclassified *Davidiella* [68] were significantly increased in T2DM patients compared with healthy individuals.

Gut mycobiota dysbiosis has also been detected in individuals with obesity. Rodríguez et al. (2015) [70] evaluated differences in the gut mycobiome between obese and nonobese subjects. Notably, the genus *Mucor* and two of its species (*M. fuscus* and *M. racemosus*) were markedly decreased in obese subjects [70]. Recently, Shoukat et al. (2023) [71] reported that obese participants presented high levels of *C. albicans*, *C. kefyr*, and *Teunomyces krusei* [71]. Another study combining a culture-dependent approach and 18S sequencing suggested that the diversity of the gut mycobiome was lower in the overweight and obese groups than in the control group. The most common genera were *Paecilomyces*, *Penicillium*, *Candida*, *Aspergillus*, *Fonsecaea*, *Geotrichum*, *Trichosporon*, *Rhodotorula*, *Rhizopus*, and *Mucor* [72].

A Chinese clinical experiment revealed that patients with NAFLD exhibited distinct gut fungal dysbiosis. Compared with healthy subjects, NAFLD patients presented considerably increased abundances of the genera *Talaromyces*, *Paraphaeosphaeria*, *Lycoperdon*, *Curvularia*, *Phialemoniopsis*, *Paraboeremia*, *Sarcinomyces*, and *Cladophialophora* and substantially decreased abundances of the genera *Pseudopithomyces*, *Leptosphaeria*, and *Fusicolla* [73]. Currently, the evidence linking intestinal fungi to NAFLD in humans is limited, as liver biopsy is still considered the gold standard for diagnosing and assessing this condition. However, advancements in noninvasive techniques will pave the way for more comprehensive studies, helping to better understand the role of the gut mycobiota in NAFLD. Another study compared the gut mycobiomes of 10 nonalcoholic steatohepatitis (NASH) patients and 10 healthy controls via culture-dependent methods. Fungi have been isolated from the guts of almost all NASH patients but not from healthy subjects. *Pichia kudriavzevii*, *C. glabrata*, *C. albicans*, and *Galactomyces Geotrichum* were isolated from NASH patients and associated with NASH pathophysiology [74].

### 5.3. Dysbiosis of the Gut Mycobiome in Liver Diseases

In recent years, an increasing number of studies have shown that intestinal fungi are closely correlated with cirrhosis. Patients with cirrhosis often have disorders of intestinal fungi, and the main manifestation is a decrease in fungal species abundance. A greater relative abundance of *Candida* was observed in fecal samples from patients with cirrhosis via ITS1 sequencing [75]. However, the gut mycobiome of decompensated cirrhosis patients is characterized by an enrichment of *Saccharomyces* and significant depletion of the genera *Aspergillus*, *Penicillium*, *Auricularia*, and *Cladosporium* [76]. Alcoholic hepatitis is another liver disease that has been examined. Lang et al. (2020) [77] conducted a study on a U.S. population and reported a high abundance of *Candida* and a low prevalence of *Penicillium* in the gut mycobiome of patients [77]. Primary sclerosing cholangitis (PSC) is a chronic liver disease characterized by inflammation and scarring of the bile ducts and is, in most cases, accompanied by UC. In a French cohort, increased levels of the genus *Exophiala* and a decreased proportion of *S. cerevisiae* were observed in patients with PSC [78].

### 5.4. Dysbiosis of the Gut Mycobiome in Neurological Diseases

With emerging evidence that the gut microbiome is intricately involved in neurological disease, it is reasonable to speculate that the fungal component plays an important role, along with other members of the gut microbiome. The gut–brain axis also affects diseases such as multiple sclerosis (MS). Shah et al. (2021) [79] compared the mycobiome of healthy controls to patients with MS. Fungal diversity, especially that of *Saccharomyces* and *Aspergillus*, was greater in MS patients than in controls [79]. However, Yadav et al. (2022) [80] reported that *Saccharomyces* was less abundant in MS patients, whereas *Candida* and *Epicoccum* were prevalent [80]. The possible role of fungi has similarly been explored in other neurological disorders, such as Alzheimer’s disease. *Candida tropicalis* and *Schizophyllum commune* were detected in high proportions in Alzheimer’s disease patients, with a decrease in *Rhodotorula mucilaginosa* [81].

Gut mycobiome dysbiosis is reported in multiple other neurological disorders, where a greater abundance of *Candida* occurs in attention-deficit/hyperactivity disorders [23], current depressive episodes [82], Rett syndrome [83], and autism spectrum disorders [84]. Nevertheless, another study of the gut mycobiome in autism patients revealed that *S. cerevisiae* was the most dominant genus rather than *Candida* [85]. Specific fungal genera, such as *Botrytis*, *Kazachstania*, *Phaeoacremonium* [86], *Tremellaceae*, *Penicillium* [87], *Purpureocillium* [88], *Saccharomyces*, and *Apiotrichum* [89], are abundant in mild cognitive impairment, Parkinson’s disease, schizophrenia, and depression patients.

### 5.5. Dysbiosis of the Gut Mycobiome in Cancers

The involvement of the gut mycobiota in carcinogenesis has recently been increasingly recognized. At the class level, the abundance of *Malasseziomycetes* increased in colorectal cancer (CRC) patients, whereas the abundances of *Saccharomycetes* and *Pneumocystidomycetes* decreased [90]. Fungal species, including *Aspergillus rambellii*, *Cordyceps* sp., *Erysiphe pulchra*, *Moniliophthora perniciosa*, *Sphaerulina musiva*, and *Phytophthora capsici*, were enriched in CRC patients in the study of Lin et al. (2022) [91], whereas *Aspergillus kawachii* was depleted [91]. Gao et al. (2017) [92] reported that fecal mycobiota dysbiosis is characterized by an increased proportion of the opportunistic fungi *Trichosporon* spp. and *Malassezia* spp. in patients at different stages of the colon carcinogenesis process [92]. *Malassezia* has frequently been found to be enriched in many types of cancers, including adenoma [93], gastric cancer [94], and hepatocellular carcinoma [95]. In hematologic malignancies or disorders, *nonalbicans Candida* spp. and *C. glabrata* are abundant in patients [96]. In contrast, *Candida* is less abundant in lung adenocarcinoma patients [97].

The number and size of studies are too limited to draw any definitive conclusions, but these results reveal an association between mycobiota alterations and cancers. Although mechanistic causality studies need to be performed, these results raise the hypothesis of the role of fungi in cancer development.

### 5.6. Dysbiosis of the Gut Mycobiome in Microbial Infections

Variations in the gut mycobiome have been reported across various microbial infections. In HIV patients, increased levels of *Candida* species (e.g., *C. albicans*, *C. krusei*, and *C. tropicalis*) were observed in Nigerian [98], French [99], and Cameroonian patients [100], whereas *Aspergillus* was elevated in the study of Yin et al. (2022) [101]. In COVID-19 cases, *C. albicans* was elevated in the study of Zuo et al. (2020) [102], whereas reductions in *Aspergillus* and *Penicillium* were noted in the work of Lv et al. (2021) [103]. In cryptococcal meningitis cases in Jiangxi, China, diverse fungi were affected, with over-presentation in *Pyricularia* sp., *Rasamsonia emersonii*, and *Wallemia ichthyophaga*, whereas decreases were observed in *Ustilaginoidea virens* and *Metschnikowia aff. pulcherrima* [104]. These findings underscore disease-specific changes in gut fungal communities, reflecting their potential roles in microbial infections.

### 5.7. Dysbiosis of the Gut Mycobiome in Cardiovascular Diseases

To further investigate the role of the mycobiome in cardiovascular disease, Zou et al. (2022) [105] used ITS1–ITS2 sequencing to examine the composition of the mycobiome in fecal samples from patients with hypertension, prehypertension, and normal blood pressure. In patients with normal blood pressure, *Mortierella* is enriched, whereas *Malassezia* is increased in patients with hypertension [105]. Using a similar approach, Chen et al. (2023) [106] compared the oral and intestinal mycobiomes of patients with normal blood pressure and those with arterial hypertension. They reported a greater abundance of *Exophiala* spp. in subjects with hypertension. *Exophiala xenobiotica* and *Exophiala mesophiles* are even correlated directly with the degree of hypertension [106]. Overall, fungi are associated with the pathogenesis of hypertension. However, further studies are needed to investigate possible causal links. Xu et al. (2020) [107] studied the gut mycobiome of coronary heart disease patients complicated with NAFLD (CHD-NAFLD). The intestinal fungal microbiota in CHD-NAFLD patients shows an increase in the abundance of *Preussia*, *Xylodon*, and *Cladorrhinum* and a reduction in the abundance of *Candida glabrata* and *Ganoderma* [107].

### 5.8. Dysbiosis of the Gut Mycobiome in Other Diseases

The contribution of the gut mycobiome to gynecological diseases has also been investigated. Intrauterine adhesions are associated with an increase in *Filobasidium* and *Exophiala* [108], whereas polycystic ovary syndrome is associated with an over-representation of the genus *Saccharomyces* and a decrease in *Lentinula* and *Aspergillus* [109]. These data suggest that fungal dysbiosis may also play a role in the pathogenesis of numerous skin, genetic, and newborn disorders. Overgrowth of the genus *Rhodotorula* is related to atopic dermatitis in infants [110]. The depletion of *Schizophyllum* has been reported in knee synovitis [111]. The gut mycobiomes of Peutz–Jeghers syndrome patients [112] and extremely-low-birth-weight infants are characterized by considerable enrichment of *Candida* [34].

Two eye diseases have been studied: bacterial keratitis and uveitis. In bacterial keratitis, *Aspergillus* and *Malassezia* are prevalent, and *Mortierella*, *Rhizopus*, *Kluyveromyces*, *Embellisia*, and *Haematonectria* are depleted [113]. Like bacterial keratitis, *Aspergillus* and *Malassezia,* in addition to *Candida*, are also prevalent in uveitis patients [114].

A study analyzing stool samples from 3-month-old infants in rural Ecuador revealed an increased relative abundance of fungi in those who developed atopic wheezing. *Pichia kudriavzevii* and *Saccharomycetales* were significantly more prevalent in the atopic wheezing group [115]. Another study categorizing participants by the composition of their neonatal intestinal microbial communities revealed an increase in the relative abundance of specific fungal genera, including *Candida* and *Rhodotorula* [116]. The potential relationship between the fungal mycobiome and tuberculosis has also been explored. Han et al. (2024) [117] reported that the abundance of *Saccharomyces* increased with reduced levels of *Aspergillus* in tuberculosis patients [117].

Graft-versus-host disease is characterized by enrichment of *Candida* [118]. Celiac disease is associated with elevated *Tricholomataceae*, *Saccharomycetaceae*, *S. cerevisiae*, and *Candida* sp., with reductions in *Pichiaceae* and Pneumocystis jirovecii [119]. Rheumatoid arthritis is associated with decreases in *Pholiota*, *Scedosporium*, and *Trichosporon* [120], whereas systemic lupus erythematosus is associated with increases in *Pezizales*, *Cantharellales*, and *Pseudaleuria* [121]. In chronic kidney disease, the abundance of *Saccharomyces* increased, whereas the abundances of *Candida*, *Bjerkandera*, *Rhodotorula*, and *Ganoderma* decreased. End-stage renal disease was exacerbated by pathogenic fungi such as *Aspergillus fumigatus*, *Cladophialophora immunda*, and *Exophiala spinifera*, with a decrease in *S. cerevisiae*.

**Table 1:** Gut mycobiome signatures and dysbiosis associated with human diseases.

Human Disease		Country	Fungal Alteration	References
Gastrointestinal tract diseases	Inflammatory bowel disease	Philadelphia, USA	*Candida* ↑	[50]
	Inflammatory bowel disease	Paris, France	*Candida albicans* ↑ *Saccharomyces cerevisiae* ↓	[51]
	Inflammatory bowel disease	Japan	*Candida* ↑ *Saccharomyces* ↓ *Sarocladium* ↓	[53]
	Inflammatory bowel disease (with *Clostridioides difficile* infection)	Beijing, China	*Saccharomyces cerevisiae* ↑	[52]
	Crohn’s disease	China	*Candida albicans* ↑ *Aspergillus clavatus* ↑ *C. neoformans* ↑	[57]
	Crohn’s disease	Northern France–Belgium	*Candida tropicalis* ↑	[58]
	Crohn’s disease	China	*Candida* ↑ *Aspergillus* ↓ *Sordariomycetes* ↓ *Penicillium* ↓	[56]
	Crohn’s disease	Krakow, Poland	*Candida tropicalis* ↑ *Malassezia* spp. ↓	[59]
	Crohn’s disease	Southwest China	*Exophiala dermatitidis* ↑ *Clonostachys* ↑ *Humicola* ↑ *Lophiostoma* ↑	[61]
	Crohn’s disease	Krakow, Poland	*Candida* ↑ *Malassezia* ↑ *Debaryomyces hansenii* ↑	[60]
	Ulcerative colitis	Beijing, China	*Scytalidium* ↑ *Morchella* ↑ *Paecilomyces* ↑ *Humicola* ↓ *Wickerhamomyces* ↓	[63]
	Ulcerative colitis	Iran	*Candida albicans* ↓ *Saccharomyces cerevisiae* ↓	[62]
	Ulcerative colitis	Rome, Italy	*Piptoporus* ↑ *Candida* ↑ *Hyphodontia* ↑ *Meyerozyma* ↓ *Malassezia* ↓	[64]
	Hirschsprung-associated enterocolitis	Stockholm, Sweden California, USA	*Candida* sp. ↑ *Malassezia* ↓ *Saccharomyces* sp. ↓	[122]
	Irritable bowel syndrome	Piacenza, Italy	*Candida* spp. ↑	[54]
	Diarrhea-predominant irritable bowel syndrome	Wuhan, China	*Mycosphaerella* ↑ *Aspergillus* ↑ *Sporidiobolus* ↑ *Pandora* ↑	[55]
Liver diseases	Cirrhosis	Virginia, USA	*Candida* ↑	[75]
	Alcoholic hepatitis	USA	*Candida* ↑ *Penicillium* ↓	[77]
	Primary sclerosing cholangitis	Paris, France	*Exophiala* ↑ *Saccharomyces cerevisiae* ↓	[78]
	Decompensated cirrhosis	Beijing, China	*Saccharomyces* ↑ *Aspergillus* ↓ *Penicillium* ↓ *Auricularia* ↓ *Cladosporium* ↓	[76]
Neurological diseases	Rett syndrome	Italy	*Candida* ↑	[83]
	Autism Spectrum Disorders	Siena, Italy	*Candida* ↑	[84]
	Autism Spectrum Disorders	China	*Saccharomyces cerevisiae* ↑ *Aspergillus versicolor* ↓	[85]
	Current depressive episode	Hangzhou, China	*Candida* ↑ *Penicillium* ↓	[82]
	Mild cognitive impairment	USA	*Botrytis* ↑ *Kazachstania* ↑ *Phaeoacremonium* ↑ *Cladosporium* ↑ *Meyerozyma* ↓	[86]
	Alzheimer’s disease	Zhejiang, China	*Candida tropicalis* ↑ *Schizophyllum commune* ↑ *Rhodotorula mucilaginosa* ↓	[81]
	Multiple sclerosis	Missouri, USA	*Saccharomyces* ↑ *Aspergillus* ↑	[79]
	Multiple sclerosis	Iowa, USA	*Candida* ↑ *Epicoccum* ↑ *Saccharomyces* ↓	[80]
	Parkinson’s disease	United Kingdom	*Tremellaceae* ↑ *Penicillium* ↑ *Saccharomyces* ↓	[87]
	Schizophrenia	China	*Purpureocillium* ↑	[88]
	Attention-deficit/hyperactivity disorder	Kaohsiung, Taiwan	*Candida albicans* ↑	[23]
	Depression	China	*Saccharomyces* ↑ *Apiotrichum* ↑ *Aspergillus* ↓ *Xeromyces* ↓	[89]
Cancers	Hematologic malignancy or disorders	Istanbul, Turkey	*Non-albicans Candida* spp. ↑ *C. glabrata* ↑	[96]
	Colorectal cancer	Shanghai, China	*Trichosporon* ↑ *Malassezia* ↑	[92]
	Colorectal cancer	Hong Kong, China	*Malasseziomycetes* ↑ *Saccharomycetes* ↓ *Pneumocystidomycetes* ↓	[90]
	Colorectal cancer	China	*Aspergillus rambellii* ↑ *Cordyceps* sp. ↑ *Erysiphe pulchra* ↑ *Moniliophthora perniciosa* ↑ *Sphaerulina musiva* ↑ *Phytophthora capsici* ↑ *A. kawachii* ↓	[91]
	Gastric cancer	Nanjing, China	*Cutaneotrichosporon* ↑ *Malassezia* ↑ *Rhizopus* ↓ *Rhodotorula* ↓	[94]
	Adenoma	Shanghai, China	*Malassezia restricta* ↑ *Leucoagaricus_sp_SymCcos* ↓ *fungal_sp_ARF18* ↓	[93]
	Hepatocellular carcinoma	Wuhan, China	*Malassezia* ↑ *Malassezia* sp. ↑ *Candida* ↑ *C. albicans* ↑	[95]
	Pancreatic ductal adenocarcinoma	New York, USA	*Malassezia* spp. ↑	[123]
	Lung adenocarcinoma	Beijing, Suzhou, and Hainan, China	*Saccharomyces* ↑ *Aspergillus* ↑ *Apiotrichum* ↑ *Candida* ↓	[97]
Microbial infections	HIV	Ile-Ife, Nigeria	*Candida albicans* ↑ *Candida krusei* ↑ *Candida tropicalis* ↑	[98]
	HIV	Marseille, France	*Candida albicans* ↑ *Candida tropicalis* ↑	[99]
	HIV	Southwest Cameroon	*Candida* ↑	[100]
	HIV	China	*Aspergillus* ↑	[101]
	COVID-19	Hong Kong, China	*Candia albicans* ↑	[102]
	COVID-19	Hangzhou, China	*Aspergillus* ↓ *Penicillium* ↓	[103]
	H1N1	Hangzhou, China	*Candida glabrata* ↑ *Aspergillus* ↓ *Penicillium* ↓	[103]
	Cryptococcal meningitis	Jiangxi, China	*Pyricularia* sp. ↑ *Rasamsonia emersonii* ↑ *Cytospora leucostoma* ↑ *Wallemia ichthyophaga* ↑ *Ustilaginoidea virens* ↓ *Metschnikowia aff. pulcherrima* ↓ *Pyricularia pennisetigena* ↓ *Jimgerdemannia flammicorona* ↓	[104]
Eye diseases	Bacterial Keratitis	Telangana, India	*Aspergillus* ↑ *Malassezia* ↑ *Mortierella* ↓ *Rhizopus* ↓ *Kluyveromyces* ↓ *Embellisia* ↓ *Haematonectria* ↓	[113]
	Uveitis	Hyderabad, India	*Malassezia restricta* ↑ *Candida albicans* ↑ *Candida glabrata* ↑ *Aspergillus gracilis* ↑	[114]
Metabolic diseases	Type 1 Diabetes	Istanbul, Turkey	*Candida albicans* ↑	[124]
	Type 1 Diabetes	Finland	*Saccharomyces* ↑ *Candida* ↑	[55]
	Type 1 Diabetes	Krakow, Poland	*Saccharomyces* ↓	[66]
	Type 2 Diabetes	Pune, India	*Aspergillus* ↑ *Candida* ↑	[69]
	Type 2 Diabetes	Hyderabad, India	*Candida* ↑ *Agaricus* ↓ *Chlorophyllum* ↓ *Coprinopsis* ↓ *Leucoagaricus* ↓ *Termitomyces* ↓ *Trametes* ↓ *Trichoderma* ↓ *Volvariella* ↓	[67]
	Type 2 Diabetes	Sharjah, United Arab Emirates	*Malessezia firfur* ↑ Unclassified *Davidiella* ↑ Unclassified *Basidiomycota* ↓	[68]
	Obesity	Girona, Spain	*Mucor racemosus* ↓ *M. fuscus* ↓	[70]
	Obesity	Juiz de Fora, Brazil	*Paecilomyces* sp. ↑ *Penicillium* sp. ↑ *Candida* sp. ↑ *Aspergillus* sp. ↑ *Fonsecaea* sp. ↑ *Geotrichum* sp. ↑ *Trichosporon* sp. ↑ *Rhodotorula* sp. ↑ *Rhizopus* sp. ↑ *Mucor* sp. ↑	[72]
	Obesity	Mexico	*Candida* spp. ↑	[125]
	Obesity	Islamabad, Pakistan	*Candida kefyr* ↑ *C.albicans* ↑ *Teunomyces krusei* ↑	[61]
	Nonalcoholic fatty liver disease	Zhejiang, China	*Talaromyces* ↑ *Paraphaeosphaeria* ↑ *Lycoperdon* ↑ *Curvularia* ↑ *Phialemoniopsis* ↑ *Paraboeremia* ↑ *Sarcinomyces* ↑ *Cladophialophora* ↑ *Sordaria* ↑ *Leptosphaeria* ↓ *Pseudopithomyces* ↓ *Fusicolla* ↓	[73]
	Nonalcoholic Steatohepatitis	Marseille, France	*Pichia kudriavzevii* ↑ *Candida glabrata* ↑ *C. albicans* ↑ *Galactomyces geotrichum* ↑	[74]
Respiratory diseases	Atopic wheeze	Ecuador	*Pichia kudriavzevii* ↑ *Saccharomycetales* ↓	[115]
	Asthma	Michigan, USA	*Candida* ↑ *Rhodotorula* ↑	[116]
	Tuberculosis	Xinxiang, China	*Saccharomyces* ↑ *Aspergillus* ↓	[117]
Immunological diseases	Graft-versus-host disease	The Netherlands	*Candida* spp. ↑	[118]
	Celiac disease	Riyadh, Kingdom of Saudi Arabia	*Tricholomataceae* ↑ *Saccharomycetaceae* ↑ *Saccharomycetes* ↑ *Saccharomyces cerevisiae* ↑ *Candida* sp. ↑ *Pichiaceae* ↓ *Pichia kudriavzevii* ↓ *Pneumocystis* ↓ *Pneumocystis jirovecii* ↓	[119]
	Rheumatoid arthritis	Dalian, China	*Pholiota* ↓ *Scedosporium* ↓ *Trichosporon* ↓	[120]
	Systemic lupus erythematosus	China	*Pezizales* ↑ *Cantharellales* ↑ *Pseudaleuria* ↑	[121]
Kidney diseases	Chronic kidney disease	China	*Saccharomyces* ↑ *Candida* ↓ *Bjerkandera* ↓ *Rhodotorula* ↓ *Ganoderma* ↓	[126]
	End-stage renal disease	China	*Aspergillus fumigatus* ↑ *Cladophialophora immunda* ↑ *Exophiala spinifera* ↑ *Hortaea werneckii* ↑ *Trichophyton rubrum* ↑ *Saccharomyces cerevisiae* ↓	[127]
Cardiovascular diseases	Chronic Heart Failure	Italy	*Candida* ↑	[128]
	Coronary heart disease (with nonalcoholic fatty liver disease)	Beijing, China	*Preussia* ↑ *Xylodon* ↑ *Cladorrhinum* ↑ *Candida glabrata* ↓ *Ganoderma* ↓	[107]
	Hypertension	China	*Malassezia* ↑ *Mortierella* ↓	[105]
	Hypertension	Shanghai, China	*Exophiala xenobiotica* ↑ *Exophiala mesophila* ↑	[106]
Gynecological diseases	Intrauterine adhesions	China	*Filobasidium* ↑ *Exophiala* ↑	[108]
	Polycystic ovary syndrome	Jilin, China	*Saccharomyces* ↑ *Lentinula* ↑ *Aspergillus* ↓	[109]
Skin disease	Atopic dermatitis	Bangkok, Thailand	*Rhodotorula* sp. ↑ *Wickerhamomyces* sp. ↓ *Kodamaea* sp. ↓	[110]
Inflammatory disease	Knee synovitis	Hunan, China	*Schizophyllum* ↓	[111]
Genetic disease	Peutz–Jeghers syndrome	Jinan, China	*Candida* ↑	[112]
Newborn disease	Extremely-low-birth-weight infants	New York, USA	*Candida* sp. ↑ *Clavispora* sp. ↑	[34]

↑: Enrichment ↓: Depletion.

## 6. The Gut Mycobiome in Precision Medicine

The sequencing of the human genome in 2001 [129] marked a significant milestone, advancing our understanding of the genetic basis of disease while also driving the development of DNA sequencing technologies essential for translating these insights into clinical applications. This has given rise to precision genomic medicine, which tailors treatment and healthcare decisions based on an individual’s genetic makeup and the identification of specific genomic markers for disease [130]. More broadly, precision medicine integrates genetic data with information on a patient’s lifestyle, medical history, and population characteristics, utilizing clinical data and biomarkers to guide treatment strategies. Since the genome is often viewed as a fundamental determinant of human individuality—particularly in the context of disease—precision medicine is sometimes mistaken for genomic medicine. However, precision medicine extends beyond genomics to encompass downstream factors such as gene and protein expression, as well as metabolic markers. Despite this broader scope, genomic data remain the most widely used and have achieved considerable success [131]. By refining disease diagnosis and minimizing treatment risks—such as side effects and non-responsiveness to medications—precision medicine has the potential to transform healthcare. Ideally, this approach will not only enable highly individualized treatments but also shift medical practice toward cost-effective, preventive care, ultimately reducing the financial burden of healthcare while improving patient outcomes [5].

The microbiome, much like any ecological system, is remarkably complex. Despite the intricate nature of gut microbial communities, advancements in NGS and the development of sophisticated bioinformatics tools have significantly enhanced our ability to analyze and characterize the human gut microbiota [35]. As a result, the microbiome is now positioned as a promising frontier in precision medicine, with its clinical applications becoming increasingly viable [5]. To support these advancements, the National Center for Biotechnology Information (NCBI) hosts a wide range of genomic data repositories that follow a structured progression—from fundamental genomic sequences (SRA, TraceArchive, Gene, GenBank, Gene, RefSeq) to genetic variations (dbSNP, dbMHC, ClinVar, dbVar); to phenotypic associations (GEO, OMIM, ClinVar, dbGAP, PhenGenI); and finally, to clinical applications (ClinGen, GTR). These repositories serve as crucial platforms for storing, curating, annotating, standardizing, and interpreting microbiome data, enabling their integration into both population-wide and precision medicine approaches. By linking microbiome data with human genetic variation, phenotypic traits, health conditions, and disease states, these resources pave the way for the microbiome’s role in personalized healthcare [132].

The gut mycobiome is an essential contributor to host immune physiology, thereby influencing the pathogenesis and progression of various diseases. Consequently, targeting the gut mycobiome for therapeutic purposes presents an innovative and promising approach. Various mycobiome-based interventions, including fecal microbiota transplantation (FMT), antifungal and antibiotic treatments, dietary modifications, and probiotics containing both fungal and bacterial strains, have demonstrated significant potential in reshaping the gut mycobiome while delivering clinical benefits [22]. Over the past decade, the field of precision medicine has undergone remarkable advancements, largely driven by breakthroughs in metagenomics. This progress has significantly expanded our understanding of the gut mycobiome. However, knowledge of gut fungal communities remains incomplete, and there is no clear consensus on the extent to which the genetic and phenotypic diversity of the human gut mycobiome has been fully mapped. Further research is essential to unlock its full potential in personalized medicine. Given the high interindividual and intraindividual variability of the gut mycobiome, as well as the underrepresentation of populations from different continents and ethnic backgrounds, there is likely much more to uncover about this microbiome component [15].

A deeper understanding of resident mycobiomes and their interaction with host immunity, particularly in relation to disease progression at various stages, could offer opportunities for early diagnosis and more targeted treatments. Advanced patient stratification based on mycobiome profiles may lead to the development of new therapeutic strategies, while manipulating or restoring a ‘healthy’ mycobiome could provide a promising approach for precision medicine. With further technological advancements, sequencing, and analysis, point-of-care diagnostics for fungal diseases may become a reality in the future [133].

### 6.1. The Gut Mycobiome in Disease Diagnosis

Early disease detection is crucial; however, not everyone has regular access to health screenings that facilitate the identification of symptoms in their initial stages. Once a disease manifests, the next step often involves biomarker analysis to confirm its presence [134]. The gut microbiome plays a significant role in overall health, influencing different stages of disease progression throughout the body [6].

The advancement of diagnostic tests utilizing biomarkers for primary diagnosis is a fundamental aspect of precision medicine [135]. While research in this area remains limited, existing studies have demonstrated the microbiome’s involvement in human diseases and its potential as a diagnostic and therapeutic biomarker in the near future. However, these findings are still in their early stages, highlighting the necessity for more extensive in vitro and in vivo studies with confirmatory tests for each disease. This is particularly important for establishing reliable microbiome signatures, especially those related to fungi [7].

There are few follow-up cohort studies examining the link between mycobiome composition and IBD outcomes, and well-structured longitudinal prospective studies are scarce. A recent investigation into the fecal mycobiome of Norwegian IBD patients and healthy controls revealed a strong correlation between specific fungal species and disease severity, as well as the likelihood of requiring surgery [136]. This study included 89 IBD patients, 40 of whom were monitored clinically for six years after sample collection. Patients with more severe disease—characterized by the need for intensified medical intervention—exhibited higher levels of Clavispora and lower levels of Phaeococcomyces and *Penicillium* than those with a milder disease course. At the species level, severe disease progression was linked to increased abundances of *C. sake* and *Galactomyces pseudocandidus*, along with notable reductions in various *Penicillium* species. Furthermore, *C. carnescens* was significantly more prevalent in patients who required surgery during the follow-up period, whereas *C. tropicalis*, *Debaryomyces nepalensis*, and *D. hansenii* were notably reduced [136]. IBD patients who also have Clostridium difficile infection (CDI) experience significant microbial dysbiosis, notably, with an increased presence of *Saccharomyces cerevisiae*. Understanding the gut micro-ecological shifts in IBD patients with CDI could offer valuable insights into disease mechanisms and potential diagnostic approaches for this subgroup [47].

Anti-*S. cerevisiae* antibodies (ASCAs) and perinuclear antineutrophil cytoplasmic antibodies (pANCAs) are valuable diagnostic markers for differentiating CD and UC. A positive pANCA and negative ASCA profile is highly specific for UC (97% specificity), while a positive ASCA and negative pANCA profile is highly specific for CD (97% specificity), particularly with small bowel involvement. These markers can aid in the diagnosis of IBD [137].

The study of Sarrabayrouse et al. (2021) [138] explored fungal and bacterial loads as potential biomarkers for diagnosing CD and UC and predicting disease relapse. Using real-time PCR, microbial loads were analyzed in 294 stool samples, revealing significant differences between patient groups. Integrating microbial load data with demographic and laboratory data improved predictive models by 18%, achieving an area under the receiver operating characteristic curve (AUC) of 0.842 for IBD diagnosis. These findings suggest that fecal fungal and bacterial loads could serve as noninvasive biomarkers to differentiate disease subtypes and anticipate disease flares in clinical settings [138]. On the other hand, Ventin-Holmberg et al. (2021) [139] analyzed the potential of fungal components in predicting response to infliximab (IFX) therapy in IBD patients. By analyzing fecal bacterial and fungal communities before and during treatment, researchers found that non-responders had higher levels of pro-inflammatory fungi, particularly *Candida*, compared to responders. These differences in microbial composition, especially fungal taxa, suggest that the gut mycobiome could serve as a biomarker for IFX response prediction [139].

Research by Zeng et al. (2022) [61] highlighted marked differences in the intestinal fungal composition between CD patients and healthy controls (HCs), with certain fungal species potentially contributing to disease onset and perianal lesions. Specifically, *Exophiala dermatitidis* and *Candida* are linked to active disease stages [61]. Similarly, gut fungal dysbiosis and disrupted bacterial–fungal interactions have been observed in patients with diarrhea-predominant irritable bowel syndrome (D-IBS). Specific fungal taxa, including *Mycosphaerella*, *Aspergillus*, *Sporidiobolus*, and *Pandora*, are significantly correlated with IBS symptoms and have been identified as potential biomarkers for distinguishing D-IBS patients from healthy individuals [55]. Furthermore, tuberculosis patients exhibit both bacterial and fungal gut dysbiosis. A diagnostic model incorporating *Bacteroides*, *Blautia*, the *Eubacterium hallii* group, *Apiotrichum*, *Penicillium*, and *Saccharomyces* demonstrated greater diagnostic accuracy for tuberculosis than models relying on either bacterial or fungal markers alone [117].

Growing evidence suggests that gut microbiota dysbiosis plays a role in CRC and adenoma development. In a study by Gao et al. (2022) [93], 13 fungal species were identified as key biomarkers for CRC diagnosis, showing consistent associations across all analyzed samples. Among them, *Lachancea waltii* and *Phanerochaete chrysosporium* presented the strongest associations with CRC. This study introduced a promising diagnostic model that could enhance CRC treatment strategies in the future [93]. Changes in the gut mycobiome may serve as complementary tools for CRC screening, diagnosis, and prognosis, helping differentiate early disease stages from advanced disease stages. Fungal biomarkers and dysbiosis patterns have also demonstrated the ability to distinguish CRC patients from healthy individuals. A study by Coker et al. (2019) [90] highlighted significant variations in fecal fungal composition, including an increase in *Malasseziomycetes* and a decrease in *Saccharomycetes* and *Pneumocystidomycetes*, which provided strong diagnostic differentiation for CRC. Additionally, an elevated Basidiomycota-to-Ascomycota ratio was observed, further supporting the potential role of fungal biomarkers in CRC diagnosis [90]. A proteomic analysis investigating the role of the fecal microbial secretome in colorectal carcinogenesis identified distinctive fungal proteins associated with advanced-stage CRC. Specifically, *Schizosaccharomyces pombe* proteins were found to be unique to CRC patients, with four fungal proteins characterizing the advanced stage of the disease [140]. Additionally, for the first time, gut fungal profiles have been proposed as potential noninvasive biomarkers for early-stage lung adenocarcinoma diagnosis. A study by Liu et al. (2023) [97] revealed significant fungal alterations in patients with lung adenocarcinoma, where *Candida* levels were reduced, whereas *Saccharomyces*, *Aspergillus*, and *Apiotrichum* levels were elevated [97].

Yao et al. (2024) [141] explored the role of gut fungi in abdominal aortic aneurysm (AAA), a life-threatening vascular disease with limited treatment options. Using metagenomic sequencing, researchers compared the gut mycobiomes of 33 AAA patients and 31 healthy individuals, revealing significant fungal dysbiosis. AAA patients exhibited an increase in *Candida* species, and a reduction in *Saccharomyces cerevisiae*. These fungal shifts correlated with clinical indicators of AAA, suggesting their potential as diagnostic and prognostic biomarkers. Additionally, animal experiments demonstrated that *S. cerevisiae* alleviated pathological changes in AAA mice, indicating a protective role. These findings highlight the influence of gut mycobiomes on AAA progression and suggest fungal modulation as a promising therapeutic strategy, advancing vascular disease management through microbiome-based interventions [141].

Despite this evidence, it is too early to determine the real clinical significance and relevance of fungal dysbiosis and fungal biomarkers in cancer. It is of paramount importance to determine whether fungal dysbiosis actively contributes to cancer initiation and progression or if it is simply a byproduct of the disease. Further research is needed to clarify this relationship. If specific fungal signatures prove to be influential, they could pave the way for novel targeted therapies in personalized medicine, aiming to modify or restore a balanced fungal community in cancer patients [10].

### 6.2. The Gut Mycobiome in Disease Therapeutics

#### 6.2.1. Probiotics and Prebiotics

A thorough approach to diagnosing and treating an individual’s disorder could integrate genome sequencing, RNA-Seq, and metatranscriptomics. These techniques help capture microbial diversity, identify microbial gene contributions to metabolism, and uncover diagnostic targets that inform treatment strategies, including personalized or “precision” probiotics [132]. Precision probiotics consist of carefully selected microbial consortia, including commensal bacteria and bacteriophages, aimed at shifting the microbiome from a diseased to a healthy state. To develop such targeted probiotics effectively, foundational research must delve into the root causes of dysbiosis, the critical microbial species involved, and their impact on health. In addition, the development of precision probiotics requires adherence to rigorous standards for drug discovery and production. These include establishing standardized reference datasets, ensuring compliance with good manufacturing practices, and setting clear guidelines for measuring viability, identity, purity, and potency. Notably, microbiome-based probiotics have the potential to function as “companion diagnostics”, meaning that they can simultaneously detect disease markers and serve as therapeutic agents within the same test [132].

Fungal probiotics have promising anticancer properties and may play a role in both cancer prevention and treatment [10]. A probiotic blend containing *Saccharomyces*, *Lactobacillus rhamnosus*, *Lactobacillus acidophilus*, and *Bifidobacterium breve* has been found to exhibit antibiofilm and antitumor effects within the colon [142]. Another study investigated the antitumor and immunostimulatory effects of β-glucan (IS-2) purified from mutated *Saccharomyces cerevisiae*. Researchers compared its effects to β-glucan from wild-type *S. cerevisiae* in mouse models of metastatic cancer. Prophylactic and therapeutic administration of IS-2 significantly reduced lung, liver, and spleen metastasis in a dose-dependent manner and prolonged survival in tumor-bearing mice. IS-2 did not directly inhibit tumor cell growth but enhanced splenocyte proliferation and activated peritoneal macrophages to produce cytokines (IL-1β, IFN-γ, IL-12), inducing tumoricidal activity. Additionally, IS-2 boosted natural killer (NK)-cell cytotoxicity, which was essential for its antitumor effects [143].

Research by Galinari et al. (2018) [144] highlights the antioxidant and proapoptotic effects of *Kluyveromyces marxianus*, a yeast closely related to *S. cerevisiae*, which is widely used in the food industry [144]. Similarly, another study revealed that β-glucan derived from *S. cerevisiae* plays a significant role in preventing genotoxic damage [145]. In addition to *S. cerevisiae*, the probiotic yeast *S. boulardii* has been reported to limit bacterial proliferation by producing high levels of acetic acid [146]. Furthermore, *S. boulardii* has been shown to regulate inflammation and suppress CRC progression in a mouse model by inhibiting the EGFR-Mek-Erk signaling pathway. It also has proapoptotic effects on tumor cells by downregulating the expression of Akt, a key regulator of the cell cycle [147]. Overall, several fungi appear to foster an environment conducive to anticancer activity. Among them, *Schizophyllum commune*, *S. cerevisiae*, and *S. boulardii* have been recognized primarily for their antioxidant and health-promoting properties [148].

Abbas et al. (2014) [149] investigated the effects of *S. boulardii* in patients with diarrhea-dominant irritable bowel syndrome (IBS-D). In a randomized, placebo-controlled trial, *S. boulardii* supplementation significantly reduced proinflammatory cytokines (IL-8 and TNF-α) while increasing anti-inflammatory IL-10 levels and improving the IL-10/IL-12 ratio. These immunological improvements were accompanied by histological enhancements, including reduced lymphocyte and neutrophil infiltrates and improved epithelial health. Additionally, patients in the *S. boulardii* group reported greater overall quality-of-life improvements compared to the placebo group. These findings suggest *S. boulardii* as a promising adjunct therapy for IBS-D, warranting further investigation in larger trials [149]. Similarly, the study of Swidsinski et al. (2008) [150] highlights the beneficial role of *S. boulardii* in restoring gut microbiota balance in patients with chronic idiopathic diarrhea. Using fluorescence in situ hybridization, researchers observed significant dysbiosis in these patients, marked by altered bacterial composition and structural disorganization of fecal microbiota. *S. boulardii* supplementation improved key microbiota parameters, including increased levels of beneficial bacteria and normalization of fecal biostructure. These improvements correlated with a reduction in diarrheal symptoms, with 40% of patients experiencing partial relief and 30% achieving complete normalization. Notably, *S. boulardii* had no impact on the microbiota of healthy individuals, underscoring its targeted therapeutic potential for gut dysbiosis [150]. It has also been revealed that *S. boulardii* supplementation in long-term total enteral nutrition (TEN) patients increased fecal short-chain fatty acid (SCFA) levels, particularly butyrate. This effect persisted even after treatment discontinuation. Despite no significant changes in fecal flora, the increase in SCFAs, especially butyrate, may explain *S. boulardii*’s protective role against TEN-induced diarrhea [151].

*S. boulardii* showed to exert anti-inflammatory effects by producing a small, heat-stable, water-soluble factor (SAIF) that inhibits NF-κB activation and IL-8 production in intestinal epithelial cells and monocytes. *S. boulardii* supernatant suppressed IL-8 expression, prevented IκBα degradation, and reduced NF-κB-DNA binding in stimulated cells. These findings suggest that *S. boulardii* modulates host cell signaling to reduce inflammation, which may contribute to its beneficial effects in intestinal diseases [152]. Moreover, Garcia Vilela et al. (2008) [153] investigated the effect of *S. boulardii* on intestinal permeability in CD. Patients receiving *S. boulardii* showed significant improvement in mucosal barrier function, as indicated by a decrease in the lactulose/mannitol ratio over three months, whereas the placebo group saw no significant change. Despite not fully normalizing permeability, *S. boulardii* demonstrated a beneficial role in strengthening the intestinal barrier in CD patients, supporting its potential as an adjunct therapy [153]. Another study demonstrated that *S. boulardii* can improve obesity-related metabolic dysfunctions in mice. *S. boulardii* treatment reduced body weight, fat mass, hepatic steatosis, and inflammation while significantly altering gut microbiota composition. These findings suggest that *S. boulardii* may have therapeutic potential for obesity and T2DM through gut microbiota modulation [154].

Gut fungi can influence the host in both harmful and beneficial ways. Certain fungal species, such as *Candida albicans*, *Aspergillus*, and *Meyerozyma*, have been implicated in metabolic disorders such as T2DM, obesity, and NAFLD. This is primarily due to their ability to trigger immune responses and generate toxic metabolites, such as candidalysin and prostaglandin E2 (PGE2), which may contribute to disease progression. On the other hand, fungi such as *S. boulardii* and *S. cerevisiae* have demonstrated probiotic potential in alleviating metabolic disorders. Studies suggest that they can help regulate body weight, reduce liver fat accumulation, decrease inflammation, and improve blood sugar and lipid profiles [155]. Additionally, fungal genera such as Alternaria and Cochliobolus could also offer metabolic benefits. They produce bioactive metabolites—including altenusin, (S)-curvularin, dehydrocurvularin, galiellalactone, and oxacyclododecindione—that may positively influence metabolic health. Given these findings, the gut mycobiome presents an exciting avenue for potential therapeutic interventions targeting metabolic diseases [23]. However, much remains unknown about the precise mechanisms linking gut fungi to metabolic health. Future research should focus on large-scale, long-term, and multicentric studies to deepen our understanding of this complex relationship [47].

There is a clear and significant link between the gut mycobiota and brain function, highlighting its potential role in neurological and neuropsychiatric disorders. Modulating gut fungal communities may serve as a promising therapeutic approach for managing brain-related diseases. For example, supplementation with *S. boulardii* CNCM I-745 has been shown to improve intestinal neuromuscular dysfunction in a mouse model of irritable bowel syndrome before exposure to herpes simplex virus type 1 (HSV-1) [156]. Moreover, recent research has shed light on the protective effects of the gut mycobiota in mitigating inflammation within the central nervous system. In a study by Takata et al. (2015) [157], the administration of *Candida kefyr* significantly reduced the severity of experimental autoimmune encephalomyelitis, a widely used animal model for multiple sclerosis. These findings suggest that specific fungal species may play a beneficial role in neurological health and disease modulation [157].

Probiotics can be genetically modified to increase their functionality, expand their mechanisms of action, and improve their stability and integration into various formulations [158]. However, their effectiveness is influenced by interactions with an individual’s diet, pre-existing microbiota, and genetic makeup, all of which can shape both general health outcomes and the specific effects of probiotic interventions [159]. To maximize therapeutic benefits, precise patient classification and stratification are essential. Thus, a comprehensive understanding of the metagenomic potential and ecological dynamics of *Candida* probiotics is needed. Consequently, the development of precision probiotics remains a complex and challenging endeavor, necessitating rigorous research and advanced methodologies.

Rather than focusing solely on eliminating harmful microbes, an alternative approach involves enhancing the presence of beneficial microbes or modifying the microbiome’s composition and functionality in a positive manner. The compounds used for this purpose are commonly known as prebiotics. However, research on prebiotics has thus far been relatively limited in scope [160]. While this strategy shows promise as a broad-spectrum intervention for various health conditions [161], advancing precision medicine in this area will necessitate broadening the range of prebiotics under investigation. With ongoing advancements in metagenomics and metabolomics providing deeper insights into the microbiome’s metabolic potential—particularly among populations with diverse dietary patterns—there remains a critical need to identify novel prebiotic compounds that can selectively promote beneficial microbes, including fungi, to drive favorable metabolic outcomes [5].

#### 6.2.2. Dietary Interventions

The microbial composition fluctuates daily and is largely driven by meal timing and dietary choices. Personalized nutrition strategies focus on identifying specific microbiota traits that can predict an individual’s response to different diets, enabling tailored dietary interventions for disease prevention and management [162]. Gaining greater insight into how local or specialized diets influence the mycobiome is crucial, as is exploring the potential of diet as a therapeutic tool for modulating fungal communities [163]. Personalized nutrition is increasingly recognized as an effective approach to enhancing health outcomes by customizing treatments and dietary plans based on an individual’s profile [164]. These approaches aim to replace the traditional one-size-fits-all model with targeted, personalized healthcare and nutrition plans tailored to each person’s unique needs [165].

Numerous nutritional studies have explored the impact of specialized diets on the gut microbiota composition and disease progression. The Mediterranean diet (MD), characterized by the consumption of whole grains, legumes, nuts, omega-3-rich foods, olive oil, and fruits while limiting eggs and adding sugars, has been linked to a lower risk of cardiovascular disease and cancer, as well as increased longevity [166,167]. Research has highlighted its health benefits, demonstrating that a plant-based diet—rich in berries, peanuts, grapes, and seeds—promotes an anti-inflammatory gut microbiota profile, whereas a meat-based diet—high in processed meats, dairy, cheese, and cholesterol—correlates with a pro-inflammatory profile [168]. However, despite its overall positive effects, stronger adherence to the MD has also been linked to increased *C. albicans* levels and more pronounced disease symptoms [169]. A recent study revealed that short-term adherence to a Mediterranean or vegetarian diet did not result in significant changes in the gut mycobiome or reductions in inflammatory biomarkers, indicating that long-term commitment is necessary to achieve substantial health benefits [170]. Research on pediatric CD patients receiving exclusive enteral nutrition (EEN), a primary dietary treatment for this condition, revealed an inverse relationship between EEN initiation and the fecal abundances of *Candida albicans*, *Clavispora lusitaniae*, and *Cyberlindnera jadinii* [171]. Since these fungal species have been previously associated with CD development, this finding suggests that EEN may serve as an effective therapeutic strategy by positively influencing both clinical outcomes and the gut mycobiome composition.

Focusing on whole-food-based diets, nutritional studies have identified specific foods and dietary patterns that can modulate the microbiome, offering potential strategies for managing symptoms of various metabolic and inflammatory diseases. Research by Ghannoum et al. (2019) [172] demonstrated the effectiveness of a mycobiome diet in improving gastrointestinal symptoms and overall health. This diet consists of plant-based protein, a healthy fat source, and starch-rich food in every meal. Following this dietary regimen for four weeks led to notable benefits, including reduced gastrointestinal discomfort, increased energy, weight loss, and improved sleep quality. Microbial analysis of fecal samples from participants revealed an increase in beneficial fungi such as *Pichia kluyveri* and *Galactomyces Geotrichum*, alongside a reduction in *Candida* species (*C. albicans* and *C. tropicalis*), compared with pre-diet levels [172].

#### 6.2.3. Fecal Microbiota Transplantation

Fecal microbiota transplantation (FMT) is gaining attention as a treatment for diseases associated with gut dysbiosis. The approach involves transferring a healthy donor’s stool to reintroduce a stable microbial community into the gut. Most research has focused on changes in the bacterial community following FMT, with fewer studies investigating how the mycobiota varies and whether these shifts provide health benefits [26]. FMT has proven effective in treating recurrent *C. difficile* infection as an ecosystem-based approach [173]. Similar approaches have been tested in various microbiome-related diseases but have shown limited clinical success. In the case of IBD, however, FMT has highlighted the importance of donor specificity [174], indicating that individualized FMT strategies may be necessary for complex diseases such as IBD, unlike the more generalized approach used for *C. difficile* infection. In one study involving recurrent *C. difficile* infection, an overrepresentation of the fungal genus Yarrowia in recipients before FMT was linked to unsuccessful treatment outcomes [175]. These findings suggest that an imbalance in certain fungal populations in either recipients or donors may reduce the effectiveness of FMT.

While FMT has been shown to prevent bloodstream fungal infections in patients with recurrent *C. difficile* infection or UC [176], it has also revealed the presence of *C. dubliniensis* in donor feces. It remains unclear whether the donor contributes to the growth of *C. dubliniensis* in the recipient after FMT. Therefore, a thorough check for opportunistic fungi (e.g., *C. albicans*, *C. parapsilosis*, *C. tropicalis*, and *C. dubliniensis*) should be performed during donor screening in the FMT process. Overall, there is a lack of clinical and translational studies investigating the efficacy of different donor gut mycobiome configurations in treating various diseases. Additionally, the concept of tailored FMT or even fecal mycobiome transplantation, which is based on matching donor and recipient mycobiomes, should be explored more thoroughly [22].

FMT shows promise as a potential treatment for UC. In this case, FMT efficacy is linked to higher levels of *Saccharomyces* and *Aspergillus* and lower levels of *Candida* in donor feces [177]. FMT from donors enriched with beneficial fungi could also serve as a potential adjunct for enhancing the effectiveness of immunotherapy in epithelial cancers such as CRC [178]. However, more large-scale, long-term, randomized, placebo-controlled trials are needed to assess the safety, efficacy, and sustainability of these preventive and therapeutic approaches [10].

#### 6.2.4. Antifungal Drugs

Antifungal drugs are primarily used to inhibit the growth of pathogenic fungi in fungal infections. Currently, antifungal treatments are also being explored for diseases related to gut mycobiome disruptions. For example, fluconazole has been shown to effectively prevent *C. albicans* colonization in the gut of patients with UC and reduce the UC activity index in animal models [179]. Shiao et al. (2021) [180] discovered that intestinal fungi influence antitumor immune responses following radiotherapy (RT) in mouse models of breast cancer and melanoma, with fungi and bacteria exhibiting opposing effects. The removal of commensal bacteria diminished the effectiveness of RT. However, antifungal treatment mainly with fluconazole improved RT outcomes by slowing tumor growth and enhancing survival in mice. Additionally, antibiotic treatment led to an expansion of certain commensal fungi from the Saccharomycetales order, particularly increasing the presence of *Saccharomyces* and *Candida genera* [180].

While antifungals can nonspecifically target both harmful and beneficial fungi in the gut, the use of broad-spectrum antifungal medications as a treatment for nonfungal diseases remains challenging. In addition, long-term use of antifungal treatments can disrupt the gut microbiota (including both bacteria and fungi), promote the emergence of multidrug-resistant fungi, exacerbate colitis, and increase airway allergies [181]. There are also concerns about the clinical effectiveness of antifungals. Some antifungal medications are metabolized before they reach the lower gastrointestinal tract. Furthermore, the hypoxic microenvironments within filamentous fungal biofilms allow fungi to survive despite current antifungal therapies, leading to reduced efficacy. Consequently, the current antifungal approach should be refined to target specific fungi responsible for each disease [22].

## 7. The Gut Mycobiome and Precision Medicine: Challenges and Perspectives

### 7.1. Challenges and Advances in Mycobiome Research: From Methodological Hurdles to Clinical Applications of Gut Mycobiome in Disease Therapeutics

Recent research has explored the complex relationships among the gut microbiome, health, and disease, revealing intriguing but sometimes contradictory findings. The microbial profiles of different populations vary and are influenced by factors such as diet and ethnicity, highlighting the importance of considering geographical and lifestyle differences in microbiome studies [182]. For example, while previous studies have linked certain microbial species to health conditions, recent research involving more diverse populations has occasionally produced conflicting results [183]. This complexity is further increased by differences in research methodologies, including variations in DNA extraction techniques and data analysis approaches [184]. As the field progresses, it is crucial to adopt a comprehensive approach that accounts for the interactions within the entire microbial ecosystem, as well as other factors such as host genetics and diet [185]. Since microbiome research is still a rapidly advancing area of science, it often involves working at the forefront of a developing field, with inherent uncertainties that can be minimized over time through the use of more sophisticated methods and technologies [186].

Like the challenge of taxonomic classification, fungal strain differences within individuals cannot be identified using ITS metabarcoding. This sub-species variability may play a crucial role in disease onset and progression. More advanced techniques, such as Amplified Fragment Length Polymorphism (AFLP), Random Amplified Polymorphic DNA (RAPD), microsatellite typing, or whole-genome sequencing (WGS), are necessary to accurately characterize these variations. However, obtaining fungal cells from the sample of interest is a prerequisite for these methods. Culture-based techniques serve as a useful starting point for identifying fungi that can withstand the harsh conditions of the human intestine. Nevertheless, these methods often yield limited diversity, as many fungi are difficult to culture and may be overlooked [187].

A major challenge in mycobiome research is the reliance on BLAST-query results for fungal identification, which can both overestimate and underestimate diversity. Often, the closest BLAST match does not permit precise species identification, and many active fungal members of the gut microbiome are undescribed [16]. This highlights the need for improved bioinformatics tools, standardized methodologies, and expanded reference databases to increase the accuracy of mycobiome analyses. Moreover, the use of model systems, such as mice with limited and defined microbiomes, remains underutilized in fungal research despite being a mainstay for bacterial microbiome studies. Mice do not naturally harbor *Candida* species and require suppression of their native microbiome to sustain *Candida* colonization [188]. In addition, the natural gut mycobiome of laboratory mice is strongly influenced by environmental factors, making controlled studies more challenging. Existing mouse studies have focused primarily on *Candida* and disease models [189], but there is significant potential to gain deeper insights into fungal interactions—both with other microbes and with the host. As the field advances, mycologists must take the lead in unraveling the role of fungi in the human gut and throughout the body [16].

A key area for future research is the potential use of mycobiome components as rapid diagnostic markers. While they hold great promise, the diagnostic application of fungal biomarkers presents significant challenges, even in high-risk populations. In contrast, mycobiome testing could provide a comprehensive evaluation of the fungal community at a given site. However, as with all molecular-based diagnostics in mycology, there are technical hurdles to overcome, including the wide variety of fungi that need to be identified in immunocompromised patients; the need for universal sample preparation methods that account for fungal morphological differences; inconsistencies in nomenclature; and limitations in commercial platform panels, reference libraries, and databases [190].

Despite the therapeutic potential of the microbiome in general, and mycobiome in particular, its application in precision medicine requires overcoming considerable hurdles. While FMT for *C. difficile* colitis is highly effective, particularly in recurrent infections [191], the procedure remains tightly regulated. A licensed practitioner must follow an Institutional Review Board-approved protocol, and each patient must provide informed consent before undergoing therapy. Furthermore, a major limitation is our current inability to fully characterize the microbial community of donor stool samples with the necessary precision. The complexity of microbial interactions and the lack of standardized methodologies for donor screening, microbial profiling, and quality control hinder the development of optimized, reproducible microbiome-based interventions. Addressing these challenges is essential to harness the full potential of the microbiome in precision medicine. This means that we do not know the active components of the fecal transplant, and therefore, it is difficult to regulate this using standard legislation under FDA protocols. More importantly, we still do not fully understand the implications of microbiome therapy on a large scale. While fecal transplants are becoming extremely numerous with few legitimate side effects, it is still difficult to predict outcomes across a broad population. The same is true for genomic medicine, whereby the interaction of genes with the environment is difficult to predict [192]. This requires enormous sample populations for any investigation to be statistically significant. Although the future is bright for genomic medicine, particular issues currently impede efforts toward its development [5].

The number of publications related to the microbiome has increased in recent years, with an increasing focus on the mycobiome [193]. Although the number of studies examining its association with various diseases is increasing, definitive conclusions about the clinical significance of the mycobiome are still lacking. Multiple obstacles persist, including the need for validation and standardization of sampling and analysis methods, which should be explored in larger studies. At present, no standardized approach exists for mycobiome sequencing, with variations in DNA extraction, primer selection, sequencing techniques, reference databases, and analytical methods hindering reliable meta-analyses of clinical studies [194].

Furthermore, the causal or consequential relationship between gut mycobiome alterations and disease is largely unclear and remains to be established in the future. Gut fungi may act as either drivers or passengers of disease, depending on the specific condition. For example, *Candida albicans* has been consistently found at elevated levels in patients with various diseases, potentially as a result of antibiotic use or disruptions in the gut microbiome balance. Additionally, variations in the strain diversity and molecular characteristics of a single fungal species across individuals may lead to different phenotypic and therapeutic responses. Given the many uncertainties surrounding the role of gut fungi in disease development and treatment, further research is essential to bridge the gap between mycobiome studies and clinical applications [22]. For example, research on cancer-associated mycobiomes should go beyond identifying correlations and focus on establishing causation, fostering collaboration, and conducting mechanistic studies. While correlation studies are valuable for guiding causal investigations, researchers must avoid overstating conclusions without thorough experimental validation. Demonstrating a direct link between fungi and cancer phenotypes remains a major challenge, requiring controlled experiments and rigorous testing to confirm that specific fungi are both present and actively involved in tumor development or progression. The use of animal models, such as germ-free mice, can help replicate cancer conditions and assess the role of entire microbial communities, specific fungal–bacterial interactions, or individual fungi [195].

Manipulating the gut mycobiome presents several ethical challenges that must be addressed as research and therapeutic applications evolve. One major concern is informed consent and autonomy, as interventions targeting the gut mycobiome may have unforeseen consequences. Participants must be fully aware of potential risks and benefits to make informed decisions [196]. Privacy and data protection are also critical, given the sensitivity of microbiome data. Ethical research requires stringent safeguards to prevent unauthorized access or misuse of personal information [197]. Additionally, the long-term impact and unintended consequences of altering the gut mycobiome must be considered, as such modifications could affect personal health and disease susceptibility. Ethical oversight is necessary to monitor and mitigate any adverse effects

### 7.2. Integration of Mycobiome Data with Omics Data

Beyond technical expertise, interpreting multi-omics data from a biological perspective remains a significant hurdle. Identifying which microbial species generate specific metabolites linked to health and disease is still unclear. To make research findings applicable for therapeutic development, a deeper understanding of metabolite origins and their potential pleiotropic effects is fundamental. Addressing these challenges requires advanced analytical frameworks capable of integrating and processing multiple types of omic data. Future research may need to incorporate multiple omic techniques rather than relying on just one or two, compensating for the limitations of individual methods. This integrative approach represents a key step toward advancing precision medicine and nutrition while enhancing our understanding of how the gut mycobiota functions in health and disease [40]. While the impact of antimicrobials such as antibiotics on fungal composition is well established, the effects of modifying the mycobiome with antifungals or specific diets on bacterial populations are less understood. An integrative approach utilizing systems biology could provide valuable insights into the trans-kingdom network (including bacteria, fungi, viruses, and archaea) and its influence on immune responses in the human gut. Future efforts should focus on addressing these gaps to integrate the role of fungi in diseases into clinical practice [39]. This will require collaboration among mycologists, bacteriologists, immunologists, and clinicians to lay the groundwork for personalized microbiome medicine. Gaining a heightened understanding of the interactions between fungi, bacteria, and the host will help identify at-risk patients and enable more effective patient care through targeted manipulation of the microbiota [198].

Multi-omics integration provides a more comprehensive understanding of host–microbe interactions. Standardization of sample preparation, cost-effective analysis techniques, and improved bioinformatics pipelines will be key to expanding these approaches. Long-read sequencing and metaproteomics can enhance fungal community profiling, while integrating mycobiome data with other omics (e.g., genomics, transcriptomics, metabolomics) can improve patient-level insights. However, challenges remain, including cost barriers; the need for large, well-powered studies; and the complexity of data interpretation. Increased automation, machine learning, and interdisciplinary collaborations will help overcome these limitations and enhance the clinical relevance of mycobiome research [199]. The study of Scanu et al. (2024) [200] exemplifies the power of integrative multi-omic analysis in understanding UC by combining metataxonomics (ITS2 and 16S rRNA sequencing) with metabolomics (GC–MS/SPME). By analyzing fungal, bacterial, and metabolic profiles in stool samples from UC patients and healthy controls, researchers identified distinct microbial and metabolic signatures associated with UC. The integration of these multi-omic datasets confirmed a unique UC-specific gut microbiota composition and revealed interkingdom interactions between bacteria and fungi through network analysis. This approach underscores the importance of multi-omic strategies in comprehensively characterizing gut dysbiosis and microbial interactions in complex diseases like UC [200]. Another study provides a multi-omics insight into how Porphyromonas gingivalis administration alters the gut mycobiome, demonstrating distinct fungal composition changes and their correlation with bacterial communities and serum metabolites. Metagenomic and Kyoto Encyclopedia of Genes and Genomes (KEGG) analyses revealed shifts in fungal diversity and enrichment of metabolic pathways, particularly those related to lipid and tryptophan metabolism. By integrating fungal, bacterial, and metabolomic data, this research highlights the complex fungi–bacteria–metabolite interactions in gut microecology, offering a comprehensive perspective on gut mycobiome remodeling driven by an oral pathogen [201]. Multi-omics approaches, including ITS2 sequencing, metagenomics, metabolomics, and proteomics, were also used to study the gut mycobiome in males with prediabetes undergoing exercise intervention. These techniques revealed that exercise significantly alters fungal diversity and composition, linking specific fungal genera to metabolic benefits. Furthermore, integrating multi-omics data helped establish associations between fungal changes and metabolic phenotypes, bacterial microbiome shifts, and circulating metabolites [202]. Likewise, the multi-omics study by Shuai et al. (2022) [203] revealed that gut fungal composition is shaped by age, diet, and host physiology, with fungal–bacterial interactions influencing metabolic health. Dairy consumption affected *Saccharomyces* and *Candida*, while *Pichia* impacted cholesterol via bacterial functions and metabolites, highlighting fungi’s role in gut ecology and metabolism [203].

The potential for personalized healthcare is becoming increasingly attainable as research in this field progresses. Although various software tools are available for multi-omics studies aimed at understanding diseases in clinical contexts, the outputs they generate are often not easily interpretable by clinicians. These tools typically require advanced technical knowledge to ensure accurate analysis and reliable results. Future advancements in multi-omics and machine learning would benefit from a multidisciplinary approach to improve the presentation of findings in a way that supports evidence-based medical decisions. The development of more accessible reporting mechanisms could help maximize the potential of multi-omics research in uncovering insights into the gut microbiome and advancing precision medicine. Overcoming these challenges will be essential for making these technologies widely applicable and beneficial to a larger audience [6].

Machine learning (ML) techniques, including microbiome composition analysis and identification of potential therapeutic targets, are being applied in gut microbiome research [204]. Since each individual’s gut microbiome profile is unique, much like a fingerprint, ML-based approaches—such as personalized gut mycobiome reports—can support clinicians in tailoring treatment strategies [205]. ML can also be used to recognize specific patterns in a patient’s mycobiome, aiding in the early detection of diseases. It is currently being implemented to identify early signs of conditions such as cardiovascular disease, liver disease, and T1DM and T2DM [6].

### 7.3. Pharmacomicrobiomics

Variability in individual drug responses has long been recognized as a significant challenge, as it affects treatment effectiveness, poses risks to patients, and contributes to economic burdens. Several factors influence drug action, including age, disease state, concurrent medications, genetic makeup, organ function, and drug interactions [206]. More recently, the gut microbiota has emerged as a key factor in drug efficacy, particularly for orally administered drugs that undergo extensive modification by microbial enzymes [207]. The interaction between drugs and gut microbes is highly complex, with microbial metabolism often involving reduction or hydrolysis, although the specific enzymes responsible for these processes remain largely unidentified. As the role of the gut microbiota in human health becomes clearer, new research fields, including pharmacomicrobiomics, have developed. This discipline explores the impact of microbes on drug metabolism, response, and distribution [208]. Increasing evidence suggests that the gut microbiota can influence a drug’s pharmacodynamics by modifying its structure or affecting the host’s immune system and metabolism [186].

The gut mycobiome plays a vital role in drug metabolism by modifying the chemical structure and bioactivity of pharmaceuticals and natural compounds through biotransformation. Fungi use diverse enzymatic systems to metabolize xenobiotics, pharmaceuticals, and natural compounds, influencing their efficacy and safety [209].

Fungal cytochrome P450 (CYP) enzymes exhibit broad substrate specificity, enabling the conversion of complex molecules into metabolites with altered pharmacological properties. For instance, *Pleurotus ostreatus* uses CYP monooxygenases to hydroxylate aromatic drugs like carbamazepine, similar to mammalian liver metabolism. Additionally, *Pichia anomala* converts curcumin into hexahydrocurcumin and octahydrocurcumin, which have enhanced antitumor activity. Fungal transformation also impacts other bioactive compounds—*Mucor spinosus* modifies ginsenoside Rg1 into metabolites with superior anticancer properties, while *Aspergillus* species biotransform dihydrocapsaicin into inhibitors of lysine-specific demethylase 1, influencing epigenetic regulation. Furthermore, *Aspergillus niger* enhances the anti-inflammatory effects of glycyrrhetinic acid, and *Rhizopus* microsporus produces chenodeoxycholic acid metabolites that interfere with bile acid metabolism and glucose homeostasis, potentially affecting metabolic health. Beyond modifying drug efficacy, *Saccharomyces cerevisiae* contribute to pharmaceutical synthesis by performing stereoselective transformations, aiding in the production of statin intermediates. These fungal-mediated processes influence pharmacokinetics, therapeutic outcomes, and drug–microbe interactions, underlining the need for deeper exploration of fungal metabolic pathways for personalized medicine and novel drug development [209].

### 7.4. Gut Mycobiome Research in Africa: Obstacles and Opportunities

It is essential to avoid making inappropriate or unfounded associations between microbiome variations and minority status [210]. Historically, a lack of cultural awareness and disparities in access to healthcare have contributed to flawed research practices, which remain major issues in advancing precision medicine. For this field to be truly effective, it must actively include and benefit underserved populations. Mycobiome research has the potential to drive impactful clinical interventions for these communities, potentially aiding their healthcare development [5]. Notably, the 1000 Genomes Project revealed that African genomes contain 25% more genetic variants than those of other ethnic groups [211]. Despite this important discovery, fewer than 2% of the analyzed genomic datasets come from African populations. As a result, while precision medicine continues to advance, many of its most sophisticated interventions are still based primarily on well-characterized genomic data from individuals of Caucasian ancestry. This lack of representation fails to capture global genetic diversity, particularly, the vast diversity within African populations [212]. Consequently, genetic variants that are more prevalent in developing countries are under-researched, leading to poorer health outcomes and reinforcing historical healthcare disparities across Africa. With the microbiome regarded as the “second genome”, there is a renewed opportunity for African researchers to take the lead in shaping the continent’s healthcare landscape. By prioritizing the development of technologies and datasets that align with Africa’s health priorities, researchers can ensure that microbiome science benefits local populations. Unlike the early days of the genomic medicine revolution, when Africa had limited access to advanced tools, the continent is now in a stronger position. The improved availability of NGS technologies, enhanced research infrastructure, and increasing expertise in bioinformatics and microbiome science across various African institutions provide a solid foundation to meet this challenge successfully [213]. Several initiatives have recently been launched in Africa to investigate the role of the microbiome in human health and disease across continents [214]. One notable example is the South African Microbiome Initiative in Neuroscience, located at Stellenbosch University in South Africa. This pioneering project focuses on the gut–brain axis and examines how the gut microbiome affects neurocognitive health in South Africa, particularly concerning conditions such as post-traumatic stress disorder [213].

The Human Heredity and Health in Africa (H3Africa) initiative was established to strengthen the research capacity across African institutions and support precision medicine tailored to African populations. Recognizing the significance of microbial interactions with human health, H3Africa has facilitated multiple microbiome research projects. Among these initiatives is the African Collaborative Center for Microbiome and Genomics Research (ACCME) in Nigeria. Another project, the Respiratory Microbiota of African Children (ReMAC), is based at the University of Cape Town in South Africa [213]. Additionally, the South African Council for Scientific and Industrial Research (CSIR), in partnership with the Sydney Brenner Institute for Molecular Bioscience at the University of the Witwatersrand, has launched the CSIR Microbiome Mapping Initiative (CMMI). This project integrates machine learning, third-generation sequencing, environmental modeling, and bioinformatics to explore connections between gut health and more than 100 lifestyle, health, and environmental factors in South Africa [215].

## 8. Conclusions

The gut mycobiome, though a minor component of the gastrointestinal microbiota, plays a crucial role in maintaining human health and contributes to various disease states. Advances in sequencing technologies, omics approaches, and computational tools have significantly improved our understanding of fungal diversity, function, and interactions within the gut environment. Fungal dysbiosis has been implicated in a range of diseases, from gastrointestinal and metabolic disorders to neurological and cardiovascular conditions, highlighting its importance in host health. As research progresses, the potential of the gut mycobiome as a diagnostic and therapeutic target in precision medicine is becoming increasingly evident. Emerging therapeutic interventions, including probiotics, dietary modifications, fecal microbiota transplantation, and antifungal treatments, offer promising avenues for restoring the gut mycobiome balance. However, challenges remain in standardizing methodologies, establishing causal relationships, and translating findings into clinical practice. Future research should focus on refining analytical techniques, integrating fungal data into broader microbiome studies, and exploring personalized treatment strategies. A deeper understanding of the gut mycobiome could provide novel insights into disease mechanisms and enhance the precision medicine paradigm.

The gut mycobiome is emerging as a key player in precision medicine, providing a novel perspective for unraveling complex disease mechanisms and developing highly targeted interventions. Rather than serving solely as biomarkers, its distinct fungal signatures actively contribute to shaping diagnostic, prognostic, and therapeutic advancements across various diseases. By combining mycobiome research with multi-omics and personalized clinical data, medicine is shifting from broad population-based approaches to truly individualized care. Leveraging this potential could revolutionize healthcare, leading to earlier disease detection, more precise treatments, and improved patient-centered outcomes.
